# The dynamics of the metabolism of acetate and bicarbonate associated with use of hemodialysates in the ABChD trial: a phase IV, prospective, single center, single blind, randomized, cross-over, two week investigation

**DOI:** 10.1186/s12882-017-0683-6

**Published:** 2017-08-29

**Authors:** William B. Smith, Sandy Gibson, George E. Newman, Kendra S. Hendon, Margarita Askelson, James Zhao, Jamil Hantash, Brigid Flanagan, John W. Larkin, Len A. Usvyat, Ravi I. Thadhani, Franklin W. Maddux

**Affiliations:** 1 Volunteer Research Group and New Orleans Center for Clinical Research at the University of Tennessee Medical Center, 1928 Alcoa Highway, Suite 107, Knoxville, TN 37920 USA; 2 Knoxville Kidney Center, PLLC, 320 Park 40 N Blvd, Knoxville, TN 37923 USA; 3 EDETEK, 500 College Road East, Suite 200, Princeton, NJ 08540 USA; 4 Tandem Labs, 115 Silvia Street, West Trenton, NJ 08628 USA; 5 Frenova Renal Research, 920 Winter Street, Waltham, MA 02451 USA; 6 Fresenius Medical Care North America, 920 Winter Street, Waltham, MA 02451 USA; 7 Massachusetts General Hospital Division of Nephrology, 55 Fruit Street #1008, Boston, MA 02114 USA

**Keywords:** Hemodialysis, Dialysate, Bicarbonate, Acetate, Acid-Base, Hemodynamics, Metabolism

## Abstract

**Background:**

In the United States, hemodialysis (HD) is generally performed via a bicarbonate dialysate. It is not known if small amounts of acid used in dialysate to buffer the bicarbonate can meaningfully contribute to overall buffering administered during HD. We aimed to investigate the metabolism of acetate with use of two different acid buffer concentrates and determine if it effects blood bicarbonate concentrations in HD patients.

**Methods:**

The Acid-Base Composition with use of hemoDialysates (ABChD) trial was a Phase IV, prospective, single blind, randomized, cross-over, 2 week investigation of peridialytic dynamics of acetate and bicarbonate associated with use of acid buffer concentrates. Eleven prevalent HD patients participated from November 2014 to February 2015. Patients received two HD treatments, with NaturaLyte® and GranuFlo® acid concentrates containing 4 and 8 mEq/L of acetate, respectively. Dialysate order was chosen in a random fashion. The endpoint was to characterize the dynamics of acetate received and metabolized during hemodialysis, and how it effects overall bicarbonate concentrations in the blood and dialysate. Acetate and bicarbonate concentrations were assessed before, at 8 time points during, and 6 time points after the completion of HD.

**Results:**

Data from 20 HD treatments for 11 patients (10 NaturaLyte® and 10 GranuFlo®) was analyzed. Cumulative trajectories of arterialized acetate were unique between NaturaLyte® and GranuFlo® (*p* = 0.003), yet individual time points demonstrated overlap without remarkable differences. Arterialized and venous blood bicarbonate concentrations were similar at HD initiation, but by 240 min into dialysis, mean arterialized bicarbonate concentrations were 30.2 (SD ± 4.16) mEq/L in GranuFlo® and 28.8 (SD ± 4.26) mEq/L in NaturaLyte®. Regardless of acid buffer concentrate, arterial blood bicarbonate was primarily dictated by the prescribed bicarbonate level. Subjects tolerated HD with both acid buffer concentrates without experiencing any related adverse events.

**Conclusions:**

A small fraction of acetate was delivered to HD patients with use of NaturaLyte® and GranuFlo® acid buffers; the majority of acetate received was observed to be rapidly metabolized and cleared from the circulation. Blood bicarbonate concentrations appear to be determined mainly by the prescribed concentration of bicarbonate.

**Trial registration:**

This trial was registered on ClinicalTrials.gov on 11 Dec 2014 (NCT02334267).

**Electronic supplementary material:**

The online version of this article (doi:10.1186/s12882-017-0683-6) contains supplementary material, which is available to authorized users.

## Background

In end stage renal disease (ESRD), the body lacks the ability to effectively excrete its acid load, and thus hemodialysis (HD) therapy assumes an important role in regulation of acid-base homeostasis by providing base from the dialysate to neutralize acids produced from catabolism of proteins [1]. Bicarbonate dialysates are commonly utilized to buffer acidic anions [1] and prescriptions are recommended by Kidney Disease Outcomes Quality Initiative (KDOQI) guidelines to be individually titrated to achieve a predialysis blood bicarbonate concentration of ≥22 mEq/L [2]. If dialysate bicarbonate concentrations are not prescribed properly, metabolic acidosis or metabolic alkalosis may occur, which have been associated with increased risk for morbidity and mortality [3–7].

In the absence of liver disease, precursors of bicarbonate (e.g. acetate, lactate, citrate) are rapidly metabolized by the liver and other tissues into bicarbonate and used for regulation of acid-base homeostasis. However, it is currently not established to what extent dialysate acid buffer concentrates that include metabolic precursors of bicarbonate contribute to the overall bicarbonate administered and subsequent blood bicarbonate concentrations. Current practice and labeling of products denote that metabolic precursors of bicarbonate should be considered to contribute to the buffer administered and are typically added to the ordered bicarbonate dose, yielding a “total buffer” prescribed.

The aims of the Acid-Base Composition with use of hemoDialysates (ABChD) trial was to use two commonly used acid dialysate buffers to: 1. study the pragmatic dynamics of the levels of acetate patients receive from the acid dialysate during HD; 2. establish the extent to which acetate is metabolized into bicarbonate, and 3. characterize the overall peridialytic dynamics of blood bicarbonate concentrations. In this study, we used two commonly used bicarbonate dialysate types containing differing levels of an acetate buffer; acetate free buffers were not investigated.

## Methods

### Study design

The ABChD trial was a Phase IV, prospective, single center, single blind, randomized, cross-over, 2 week physiologic investigation of peridialytic acid-base dynamics and metabolism associated with use of two commercial acid buffered hemodialysates; 1. NaturaLyte®, containing 4 mEq/L of acetate, and 2. GranuFlo®, containing 8 mEq/L of acetate (refer to Table 1 for a detail of the composition of the acid concentrates). This study was conducted at Volunteer Research Group and New Orleans Center for Clinical Research located at 1928 Alcoa Highway, Suite 107, Knoxville, TN 37920 and was performed in accordance with the Declaration of Helsinki under a protocol approved by Crescent City Institutional Review Board (IRB) located at 2820 Canal Street, New Orleans, LA 70119 (IRB Protocol # 14–034). All patients provided informed consent prior to study participation, which included consent for the publication of unidentifiable patient data. This trial was publically registered with the ClinicalTrials.gov Registry Identifier: NCT02334267.

**Table 1:** Composition of NaturaLyte® and GranuFlo® acid concentrates

Acid concentrate Types (Catalog Number)	NaturaLyte (08–2251-0)	GranuFlo (0FD2251-3B)
Sodium (mEq/L)	100	100
Potassium (mEq/L)	2	2
Calcium (mEq/L)	2.5	2.5
Magnesium (mEq/L)	1	1
Acetate (mEq/L)	4	8
Chloride (mEq/L)	105.5	101.5
Dextrose (mg/dL)	100	100

Acid concentrates were selected based on each individual patient’s standard of care hemodialysis prescription. One patient was prescribed NaturaLyte with a potassium bath of 3 mEq/L (08–3251-9)

Acid concentrate constituents are expressed as acid portion only prior to the addition and reaction of NaturaLyte bicarbonate powder 4000 series or equivalent. For use with hemodialysis equipment capable of calibration for a mix ratio of 1:44 (also expressed as 45X or 1:1.72:42.28)

Source: http://www.fmcna-concentrates.com/best_practices.html

The trial randomized 11 prevalent HD patients from November 2014 to February 2015 in the Knoxville, Tennessee area of the United States. Patients were scheduled to receive two study HD treatments 7 ± 2 days apart with one non-dialysis day separating the preceding HD session. Between study-related treatments patients received HD at their usual outpatient dialysis centers. The order of acid buffer concentrate used for the dialysate preparation was randomly assigned in an overall equal allocation ratio. Patients in Group 1 received GranuFlo® and then NaturaLyte® at weekly study visits, and Group 2 had the reverse ordering. All study related HD treatments and procedures were performed in a clinical research center accredited by the United States Food and Drug Administration (FDA). Patients and laboratories were blinded from group and dialysate assignments.

#### Outcomes

The endpoints for this investigation are as follows:

1. Establishment of bicarbonate and acetate levels in the blood and dialysate before, during and after HD when using NaturaLyte® and GranuFlo® acid dialysate compositions.
2. Investigation of the associations during and after HD in blood bicarbonate increases using NaturaLyte® and GranuFlo® acid dialysate compositions.

### Patient population

Patients included in this trial were adults (age ≥ 18) with end stage renal disease (ESRD) treated by HD at a Fresenius Medical Care (FMC) clinic for > 90 days using an arteriovenous fistula (AVF) or graft (AVG), considered clinically stable (mean monthly spKt/V of ≥ 1.2; Hgb ≥ 9.0 g/dL; mean 2 week interdialytic weight gain ≤ 3.5 kg) and with no changes in vascular access and dialysis prescription (including heparin dosing) within the month prior to and throughout the trial. Patients had no changes in any phosphate binders, calcium supplements, non-dialysis related anticoagulant therapies, non-dialysate sodium bicarbonate, and/or citrate based concomitant medications 2 weeks prior to and throughout the study. Patients on systemic glucocorticoid/corticosteroids for treatment of controlled non-acute disease states (e.g. systemic lupus erythematosus) had no dose changes ≥ 2 months before study participation and throughout the trial.

Study exclusion criteria included patients that were pregnant or lactating, who missed a scheduled dialysis treatment 2 weeks prior to screening, exhibited clinically abnormal liver function tests (i.e. aspartate aminotranferase, alanine transaminase, and alkaline phosphatase ≥ 2 times the upper limit of normal, or total bilirubin > 1.9 mg/dL), had clinically significant uncontrolled blood pressure, an active/recent bleeding disorder, platelet count < 100,000, requirement of chronic supplemental oxygen use, active and significant chronic obstructive pulmonary disease, significant residual renal function, active or recent malignancy within 5 years (with exceptions for basal and squamous cell carcinoma), active and clinically uncontrolled autoimmune disease, human immunodeficiency virus, congestive heart failure (New York Heart Association class III or IV), planned surgical procedures during the study, current/recent illicit drug use or alcohol abuse, or treatment with an investigational drug, device or other interventions within 30 days prior to and during trial participation.

#### Screening

After signing informed consent, study candidates had all screening procedures conducted and eligibility assessed within ≤45 days. Inclusion and exclusion criteria was assessed for each study candidate after a physical examination with assessment of congestive heart failure via New York Heart Association classification [8], review and reconciliation of the study candidates’ concomitant medications and ancillary orders, medical history, symptoms and complaints, as well as, based on records for hemodialysis prescriptions and screening laboratories. Screening laboratory specimens were collected at the clinical research facility and included tests for a comprehensive metabolic panel, a complete blood count, and a serum pregnancy test in all women of childbearing potential. The subject most recent spKt/V was used for determining eligibility.

#### Randomization

Study participants determined to meet eligibility criteria were randomized in a 1:1 ratio to receive one of the two study HD treatment sequences (Group 1: Week 1 NaturaLyte® and Week 2 GranuFlo®; and Group 2: Week 1 GranuFlo® and Week 2 NaturaLyte®). The sequence generation for the randomized allocation of patients was performed by EDETEK using the Panther Interactive Web Response System (IWRS) based on permuted-block method with a block size of 4. The investigator’s clinical staff performed the enrollment of patients via the IWRS and then assigned patients to study HD treatments. Upon randomization, subjects were assigned a unique randomization number, which was used to blind the patients and laboratories from the assignment of the study group and dialysate buffer sequence. The randomization assignments were issued ≤ 7 days before the Week 1 interventional study visit.

#### Blinding

This protocol was single blinded, whereby the study patients and laboratories were blinded from the study group assignment and the dialysate buffer type utilized at the two study HD treatments. To ensure that the study patients were blinded to the dialysate buffer, the preparation containers were labeled in a blinded manner and patients were not disclosed their study group assignment. Laboratories were blinded to the study group and dialysate buffer type via a blinded version of the study protocol and subject and study visit numbering assignments that did not disclose the group or dialysate buffer type utilized for the study HD treatments. The study investigator was not blinded to the patients’ group and dialysate buffer assignments since the results of the study were based on laboratory measurements, and the investigator was not considered to have a notable potential to influence the study results.

#### Study procedures

Due to the complexity of the study procedures conducted in this protocol, the study was conducted at a Phase I clinical research facility. The night before each study HD treatment, patients were admitted to the clinical research facility prior to dinner and discharged the following day after a single HD treatment and all specimen collections. All meals and drinks during admission consisted of the same quantity and ingredients and were consumed during the same timeframe. The predefined timing of meals included: 1. dinner at 16 ± 2 h (4:00 pm to 8:00 pm) the evening before a study related HD session; 2. an after dinner snack at 13 ± 2 h (7:00 pm to 11:00 pm) the night before a study related HD session; and 3. breakfast at 2 h ± 30 min (7:30 am to 8:30 am) before a study related HD session.

Standardization of HD treatments was undertaken to approximate the patients’ usual dialysis. Fresenius Medical Care staff prepared the dialysate solutions and all patients received study treatments from dedicated Acute Services staff starting at 10:00 am (±15 min) using standardized Fresenius 2008 K dialysis machine, tubing, concentrates (NaturaLyte® bicarbonate concentrate and NaturaLyte® or GranuFlo® acid concentrates with 2.5 mEq/L of Calcium), Optiflux 160 NR dialyzer, and HD duration 240 min. The following parameters were based on each individual patient’s current HD prescription and had no changes: blood and dialysate flow rate, heparin dosing, potassium, sodium, and sodium bicarbonate concentrations, and blood flow rate (required to be ≥200 mL/min).

For each study related HD treatment, arterialized blood samples were drawn predialysis from the arterial fistula/graft needle; blood was collected via four 3cm^3^ lithium heparin plasma separator tubes and one 3cm^3^ EDTA tube. Intradialytic blood samples were drawn simultaneously from arterial and venous cannulation ports on the dialysis tubing bloodlines without altering dialysis ultrafiltration or blood flow rates at 25, 60, 90, 120, 150, 180, 210, and 240 min; blood was collected via three 3cm^3^ lithium heparin plasma separator tubes at each time point for arterialized and venous blood. For surveillance of the dialysis vascular access recirculation, intradialytic blood samples were drawn simultaneously from arterial and venous cannulation ports on the dialysis tubing bloodlines at 30 min; the ultrafiltration and blood pump was briefly turned off to perform blood collections into 3cm^3^ lithium heparin plasma separator tubes for blood urea nitrogen measurements. Postdialysis arterialized blood samples were drawn from arterial fistula/graft needle at 15, 30, 45, 60, 75, and 90 min; blood was collected via three 3cm^3^ lithium heparin plasma separator tubes at each time point. Inflow and outflow dialysate samples were simultaneously collected from the sample collection ports on the dialysate tubing lines predialysis and during dialysis without altering ultrafiltration or flow rates at 25, 60, 90, 120, 150, 180, 210, and 240 min; dialysate collections were performed via 10cm^3^ syringes at a collection rate of 10cm^3^ per 60 s. All samples were drawn within ±5 min of the scheduled times.

For definitions, the term “arterialized” blood is used to denote the blood that enters the dialyzer coming from the dialysis access (AVF/AVG) via the arterial bloodline, and is the most representative of the body’s biochemical status. The term “venous” blood is used to define the blood that is exiting the dialyzer and being returned to the patient via the venous bloodline; the venous blood is the most affected by the dialysate treatment and representative of the bicarbonate and acetate delivered to patients. The term “inflow” dialysate refers to the dialysate coming into the dialyzer and the term “outflow” dialysate denotes the dialysate coming out of the dialyzer.

#### Processing of laboratory specimens

All blood collection tubes (lithium heparin plasma separator and EDTA tubes) were inverted ≥8 times and immediately processed following collection. Blood specimens collected using lithium heparin plasma separator tubes were centrifuged. For predialysis blood samples two arterialized specimens in 3cm^3^ lithium heparin plasma separator tubes and one in the 3cm^3^ EDTA tube were immediately refrigerated at a sub-ambient temperature (~4 °C) without breaking the vacuum in the blood tubes. The other two arterial 3cm^3^ lithium heparin plasma separator tubes had plasma specimens transferred into 3cm^3^ conical vials, which were tightly capped, and then rapidly frozen on dry ice to at least −70 °C within 10 min of being centrifuged.

For the intradialytic and postdialysis sets of blood samples at each time point except 30 min of HD, one arterial and one venous (only venous for intradialytic collections) 3cm^3^ lithium heparin plasma separator tube was immediately refrigerated at a sub-ambient temperature; two arterial and one venous tube was refrigerated at a sub-ambient temperature for the 30 min of HD blood urea nitrogen specimens. Similar to before, the other two arterial and two venous (only venous for intradialytic collections) 3cm^3^ lithium heparin plasma separator tubes for had plasma specimens transferred into 3cm^3^ conical vials, which were tightly capped, and then rapidly frozen on dry ice to at least −70 °C.

For all dialysate specimen collection time points, processing occurred immediately after removing the 10cm^3^ syringe with the dialysate specimen from the dialysate sample collection port. At each time point, one inflow and one outflow specimen in the 10cm^3^ syringe were each immediately transferred with an 18 gauge needle into three 8cm^3^ yellow vacutainer conical tubes; approximately 3mLs from each inflow and outflow dialysate specimen was transferred each vacutainer tube without breaking the vacuum. For each dialysate specimen collected, two 8cm^3^ yellow vacutainer conical tubes were frozen to −70 °C within 10 min of collection, and one 8cm^3^ yellow vacutainer conical tube dialysate specimen was stored at a sub-ambient temperature until shipment on the day of collection.

The sub-ambient blood and dialysate specimens were shipped in sub-ambient shipment boxes on the day of collection to the designated central laboratory for bicarbonate measurements (Spectra Clinical Research, 8 King Road, Rockleigh, NJ 07647) and predialysis blood comprehensive metabolic panel and hematology complete blood count testing, as well as, blood urea nitrogen measurements of the 30 min of HD blood specimens (LabCorp, 1924 Alcoa Highway, Knoxville, TN 37920). The frozen plasma and dialysate specimens were shipped covered by dry ice in frozen shipping boxes on the day of collection to the designated central laboratory for acetate measurements (Tandem Labs, Inc., 115 Silvia Street, West Trenton, NJ 08628).

#### Bioanalytical procedures

Bicarbonate and acetate concentrations were measured in plasma and dialysate samples. Bicarbonate concentrations were analyzed by Spectra Clinical Research using Good Laboratory Practice (GLP) methods and a widely utilized and validated assay. Acetate concentrations were analyzed by Tandem Labs, Inc. using GLP-like methods and a commercially available colorimetric assay kit (EnzyChrom™ Kit EOAC-100, BioAssay Systems, CA, USA). The assay was validated for use in human plasma and dialysate specimens following extensive quality control measures [9–11]. Measurements for comprehensive metabolic panels, hematology complete blood count panels, and blood urea nitrogen were analyzed by the local LabCorp facility using standard methods with approved and validated assays.

#### Protocol amendments

There were two protocol amendments performed during the commencement of this trial. The protocol amendments were performed to feasibly accomplish study enrollment and clarify items in the protocol. The first protocol amendment included updating eligibility criteria for the permitted mean 2 week interdialytic weight gain before screening from ≤2 kg to ≤3.5 kg, defining actions to determine the presence of significant residual renal function when the quantity of urine output was in question, clarify parameters of the patients prescription to be captured and that remained unchanged with use of differing hemodialysate types, clarify potential intradialytic saline infusions to be captured, and extend the predefined time points to collect blood specimens postdialysis.

The second protocol amendment included extending the screening period from 30 days to 45 days, updating the requirement that patients were receiving dialysis three times per week 1 month before participation to that patients were on a stable dialysis prescription for the prior month with the stipulation that they received all scheduled outpatient dialysis treatments within 2 weeks prior to screening, updating that screening laboratories are to be collected at the study site for determining eligibility and not captured from historic values, permitting the use of glucocorticoids/corticosteroids for controlled and clinically stable autoimmune diseases, clarifying the procedures for reconciliation of concomitant medications, and defining permitted rescreening conditions for patients who missed a study HD treatment for non-clinical reasons.

#### Statistical methods

Since there have not been previous studies performed with a validated acetate assay to give us an expected reference value and variations in acetate blood levels to base an estimation upon, a formal power estimation was not performed for determining a sample size to drive *p*-values. It is notable to mention that there have been reports in the literature of normal acetate blood levels that are more than 10 fold different using differing invalidated assays [12–19]. Therefore, the sample size was chosen via medical judgment based on educated assumptions from the study team, and considerations of sample sizes utilized for other intense physiological studies with high number of measurements. We chose a predefined sample size of a minimum of 10 and up to 20 prevalent HD patients to achieve 10 subjects that completed both study HD treatments in this trial. This study design that included inpatient admission of subjects for a well-controlled study environment for collection of specimens was not considered feasible in a large population of patients, therefore we selected a cross over design, whereby patients act as their own control. Notably, this study was designed to have a very high number of sampling time points (16 arterialized and 8 venous blood draws during each treatment, as well as, 9 inflow and 9 outflow dialysate samplings per treatment), which yielded 84 data points per patient per treatment to determine the main study endpoints. The sample size with a cross-over design and the high number of collection time points was overall determined to be appropriate for assessment of the endpoints of the protocol.

The analysis of the endpoints was included data on the Per Protocol (PP) population, which included all data for patients who were administered at least one study related HD treatment, had post-interventional HD treatment bicarbonate and acetate data available, and did not meet any pre-specified protocol criteria for exclusion of study data; these analyses were performed after the completion of study procedures. The analysis of vital signs and general laboratories not related to the study endpoints were performed on the Safety Population, which included patients who had at least one HD treatment. For determination of the primary endpoint, descriptive statistics using standard methods were calculated and time plots were generated per patient for each HD treatment for bicarbonate and acetate concentrations in arterial, venous, inflow and outflow samples. The secondary endpoint was analyzed by assessing peridialytic changes, per patient per treatment using linear mixed effects models that were constructed with time and baseline predialysis values as fixed effects, and the patient as a random effect. Additionally, the slopes of the models were further investigated for excursions of bicarbonate blood concentrations. Exploratory analyses of a select cohort of the study population was performed using the same methods utilized for the entire study population; these analyses were performed due to unexpected findings in inflow dialysate acetate concentrations in some of the HD treatments for three patients that were not consistent with the known concentrations of acetate in the acid dialysate buffer types. To ensure proper dialysis access function during study HD treatments, variations in access recirculation of > 50% calculated via 30 min of HD blood urea nitrogen measurements were prespecified to be removed from the analysis. All analyses were performed using SAS software version 9.4 (100 SAS Campus Drive, Cary, NC 27513, USA).

## Results

### Patients, demographics, clinical parameters, adverse events, and study conduct

**Fig. 1:**
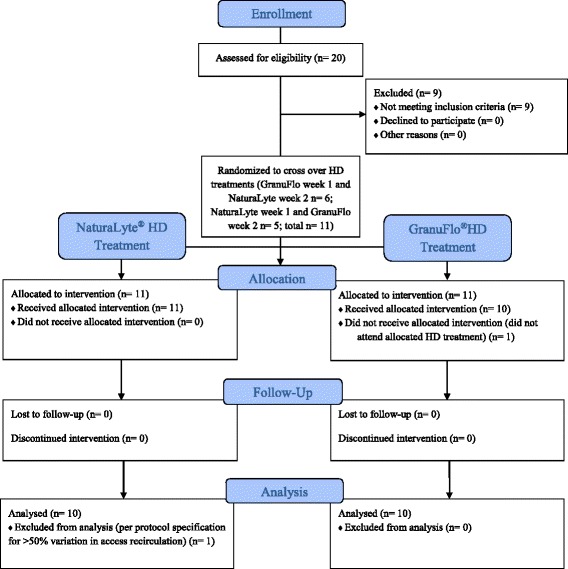
Study Patient Disposition

From November 2014 to February 2015, there were 20 patients were recruited and screened for this clinical trial and 11 patients were determined to meet eligibility criteria and were randomized to receive a single HD treatment with each of the acid buffer solutions. Six patients were randomized to receive GranuFlo® at week 1 and NaturaLyte® at week 2, and five patients were randomized to receive NaturaLyte® at week 1 and GranuFlo® at week 2. Overall, ten patients attended both treatments. During one HD treatment, one patient had > 50% variation in access recirculation, and the data was not included in the per protocol (PP) population analysis under the predefined protocol specification. Overall, there were 20 HD treatments used from the 11 patients (10 NaturaLyte® and 10 GranuFlo®) for the PP population analyses of endpoints (Fig. 1). Patients had a mean age of 53 years (standard deviation (SD) ±10), 36% were female, and 82% were African American (Table 2). The profiles of vital signs and laboratories in the Safety Population are shown Table 3 and Table 4. A detail of the blood and dialysate flow rates for each patient in the PP population is shown in Table 5. During the study no patient experienced an adverse event related to use of NaturaLyte® or GranuFlo® and there were no major protocol deviations reported.

**Table 2:** Demographics and clinical characteristics in the PP Population

Parameter	Statistic	NaturaLyte	GranuFlo	Total (All Treated)
Patient number (Per Protocol Population)	N	10	10	11
Age (years)	Mean (SD)	53.1 (10.82)	54.9 (8.77)	53.1 (10.26)
	Median	50.5	52	51
	Min, Max	35.0, 75.0	47.0, 75.0	35.0, 75.0
Sex				
Female	n (%)	3 (30.0)	4 (40.0)	4 (36.4)
Male	n (%)	7 (70.0)	6 (60.0)	7 (63.6)
Race				
American Indian or Alaska Native	n (%)	0 (0.0)	0 (0.0)	0 (0.0)
Asian	n (%)	0 (0.0)	0 (0.0)	0 (0.0)
Black	n (%)	8 (80.0)	9 (90.0)	9 (81.8)
Native Hawaiian or other Pacific Islander	n (%)	0 (0.0)	0 (0.0)	0 (0.0)
White	n (%)	2 (20.0)	1 (10.0)	2 (18.2)
Ethnic Group				
Hispanic or Latino	n (%)	0 (0.0)	0 (0.0)	0 (0.0)
Not Hispanic or Latino	n (%)	10 (100.0)	10 (100.0)	11 (100.0)
Weight (kg)	Mean (SD)	84.4 (20.35)	88.1 (23.09)	87.6 (21.99)
	Median	82.1	88.5	82.3
	Min, Max	50.5, 120.3	50.5, 120.3	50.5, 120.3
Height (cm)	Mean (SD)	164.5 (10.85)	167.4 (11.06)	166.0 (11.45)
	Median	162.7	168.3	166.4
	Min, Max	151.9, 185.9	151.9, 185.9	151.9, 185.9
BMI (kg/m^2^)	Mean (SD)	31.2 (7.32)	31.3 (7.39)	31.7 (7.12)
	Median	31.3	31.3	32.1
	Min, Max	21.9, 47.6	21.9, 47.6	21.9, 47.6

**Table 3:** Vital signs in the safety population

Parameter	Statistic	NaturaLyte	GranuFlo
Patient number (Safety Population)	N	11	10
Predialysis Systolic Blood Pressure (mmHg)	Mean (SD)	138.0 (24.50)	136.8 (22.80)
	Median	130	139.5
	Min, Max	107.0, 193.0	101.0, 170.0
Postdialysis Systolic Blood Pressure (mmHg)	Mean (SD)	119.2 (10.32)	124.3 (15.73)
	Median	121	122.5
	Min, Max	101.0, 134.0	99.0, 147.0
Predialysis Diastolic Blood Pressure (mmHg)	Mean (SD)	76.9 (11.90)	82.4 (12.54)
	Median	74	84
	Min, Max	60.0, 100.0	67.0, 106.0
Postdialysis Diastolic Blood Pressure (mmHg)	Mean (SD)	71.5 (14.26)	75.8 (9.62)
	Median	72	75
	Min, Max	47.0, 93.0	57.0, 94.0

**Table 4:** Laboratory parameters in the safety population

Parameter	Statistic	Screening	Week 1	Week 2
Patient number (Safety Population)	N	11	11	10
Erythrocytes (10^12/L)	Mean (SD)	4.0 (0.58)	3.6 (0.52)	3.4 (0.57)
	Median	4.1	3.7	3.6
	Min, Max	2.8, 4.7	2.9, 4.3	2.4, 4.0
Leukocytes (10^9/L)	Mean (SD)	8.3 (5.45)	7.1 (2.70)	5.8 (1.94)
	Median	6.9	6.4	5.6
	Min, Max	3.1, 23.9	3.7, 13.4	2.6, 8.5
Hematocrit (%)	Mean (SD)	37.5 (4.57)	34.1 (3.79)	31.7 (4.14)
	Median	37.9	34.3	31.8
	Min, Max	28.9, 43.2	28.9, 40.5	23.8, 36.4
Hemoglobin (g/dL)	Mean (SD)	11.8 (1.56)	10.9 (1.27)	10.1 (1.38)
	Median	12	10.8	10
	Min, Max	9.0, 13.8	9.3, 12.7	7.6, 11.8
MCH (pg/cell)	Mean (SD)	30.1 (2.42)	30.2 (2.35)	29.8 (2.62)
	Median	30.8	30.7	30.1
	Min, Max	23.8, 32.5	24.1, 33.1	23.5, 32.5
MCHC (g/dL)	Mean (SD)	31.6 (0.98)	32.0 (0.97)	31.8 (1.24)
	Median	31.7	32.1	31.8
	Min, Max	29.6, 33.4	29.7, 33.2	29.1, 33.3
MCV (fL)	Mean (SD)	95.2 (6.15)	94.2 (5.94)	93.7 (6.03)
	Median	95.4	94.3	93.5
	Min, Max	80.6, 104.3	80.9, 102.1	80.6, 101.3
RDW (%)	Mean (SD)	14.8 (1.36)	14.8 (1.44)	14.3 (1.09)
	Median	14.5	14.3	14
	Min, Max	12.7, 18.0	13.0, 17.9	13.3, 16.5
Platelets (10^9/L)	Mean (SD)	251.6 (117.6)	220.8 (85.90)	205.8 (97.71)
	Median	227	192	171
	Min, Max	159.0, 501.0	143.0, 396.0	101.0, 450.0
MPV (fL)	Mean (SD)	10.6 (0.84)	10.9 (1.01)	11.0 (0.94)
	Median	10.4	10.5	11.1
	Min, Max	9.5, 12.2	9.1, 12.4	9.3, 12.4
Absolute Basophils (10^9/L)	Mean (SD)	0.0 (0.04)	0.0 (0.04)	0.0 (0.03)
	Median	0	0	0
	Min, Max	0.0, 0.1	0.0, 0.1	0.0, 0.1
Absolute Eosinophils (10^9/L)	Mean (SD)	0.2 (0.13)	0.2 (0.11)	0.2 (0.11)
	Median	0.1	0.2	0.1
	Min, Max	0.1, 0.4	0.1, 0.4	0.1, 0.4
Absolute Lymphocytes (10^9/L)	Mean (SD)	2.0 (1.03)	1.7 (0.53)	1.6 (0.73)
	Median	1.7	1.7	1.6
	Min, Max	0.7, 4.3	0.9, 2.5	0.6, 2.9
Absolute Monocytes (10^9/L)	Mean (SD)	0.6 (0.38)	0.7 (0.34)	0.5 (0.18)
	Median	0.5	0.6	0.5
	Min, Max	0.3, 1.7	0.3, 1.5	0.2, 0.8
Absolute Neutrophils (10^9/L)	Mean (SD)	5.4 (4.30)	4.5 (2.45)	3.5 (1.31)
	Median	4.4	3.8	3.6
	Min, Max	2.0, 17.7	2.1, 10.5	1.7, 5.7
Percent Basophils (%)	Mean (SD)	0.4 (0.24)	0.6 (0.35)	0.6 (0.40)
	Median	0.3	0.6	0.5
	Min, Max	0.2, 1.0	0.1, 1.3	0.1, 1.5
Percent Eosinophils (%)	Mean (SD)	2.5 (1.78)	2.9 (1.35)	2.8 (1.23)
	Median	1.9	2.9	2.2
	Min, Max	0.5, 5.7	1.3, 4.9	1.7, 5.3
Percent Lymphocytes (%)	Mean (SD)	25.6 (9.02)	25.2 (7.76)	27.6 (7.32)
	Median	24.9	24.9	27.8
	Min, Max	11.7, 39.5	12.3, 36.1	15.8, 38.1
Percent Monocytes (%)	Mean (SD)	8.0 (2.47)	10.2 (5.97)	9.5 (3.26)
	Median	7	8.1	8.8
	Min, Max	5.9, 13.8	4.6, 24.8	5.2, 17.0
Percent Neutrophils (%)	Mean (SD)	63.4 (11.25)	61.1 (8.92)	59.6 (7.16)
	Median	64.5	56.9	61.5
	Min, Max	44.6, 78.9	51.2, 78.5	44.6, 67.1
Sodium (mmol/L)	Mean (SD)	141.1 (3.11)	141.0 (2.65)	141.1 (2.39)
	Median	141	141	141
	Min, Max	136.0, 149.0	138.0, 147.0	137.0, 144.0
Potassium (mmol/L)	Mean (SD)	4.8 (0.50)	4.8 (0.35)	4.5 (0.44)
	Median	4.9	4.8	4.4
	Min, Max	3.8, 5.5	4.1, 5.4	3.8, 5.2
Chloride (mmol/L)	Mean (SD)	96.2 (4.79)	98.4 (4.30)	97.5 (5.26)
	Median	96	97	99
	Min, Max	88.0, 102.0	93.0, 106.0	89.0, 106.0
BUN (mg/dL)	Mean (SD)	36.7 (16.33)	45.8 (15.06)	41.3 (17.87)
	Median	33	46	36
	Min, Max	16.0, 69.0	27.0, 83.0	20.0, 82.0
Creatinine (mg/dL)	Mean (SD)	7.0 (1.46)	8.1 (1.25)	7.9 (1.06)
	Median	6.5	7.9	8.3
	Min, Max	4.6, 9.5	6.4, 10.8	6.4, 9.4
BUN/Creatinine (RATIO)	Mean (SD)	5.2 (1.89)	5.7 (2.00)	5.0 (2.36)
	Median	5	5	4.5
	Min, Max	2.0, 9.0	3.0, 10.0	2.0, 10.0
Glucose (mg/dL)	Mean (SD)	125.1 (56.33)	139.3 (50.78)	151.9 (56.93)
	Median	89	118	130.5
	Min, Max	78.0, 223.0	84.0, 221.0	97.0, 237.0
Calcium (mg/dL)	Mean (SD)	9.3 (0.65)	8.9 (0.50)	8.7 (0.57)
	Median	9.3	9	8.8
	Min, Max	8.3, 10.8	8.0, 9.8	7.9, 9.5
Protein (g/dL)	Mean (SD)	7.4 (0.42)	6.7 (0.50)	6.5 (0.42)
	Median	7.4	6.8	6.5
	Min, Max	6.6, 8.1	6.0, 7.8	5.9, 7.5
Globulin (g/dL)	Mean (SD)	3.0 (0.34)	2.7 (0.26)	2.6 (0.25)
	Median	2.9	2.8	2.6
	Min, Max	2.3, 3.5	2.4, 3.2	2.3, 3.2
Albumin (g/dL)	Mean (SD)	4.4 (0.32)	4.0 (0.33)	3.9 (0.28)
	Median	4.3	4.1	3.9
	Min, Max	3.8, 4.9	3.4, 4.6	3.4, 4.3
A/G Ratio (RATIO)	Mean (SD)	1.5 (0.23)	1.5 (0.14)	1.5 (0.14)
	Median	1.4	1.5	1.5
	Min, Max	1.2, 2.0	1.2, 1.6	1.3, 1.7
Albumin/Total Protein (RATIO)	Mean (SD)	0.6 (0.06)	0.6 (0.00)	0.6 (0.00)
	Median	0.6	0.6	0.6
	Min, Max	0.5, 0.7	0.6, 0.6	0.6, 0.6
ALP (ukat/L)	Mean (SD)	95.4 (37.60)	88.5 (35.42)	79.2 (32.93)
	Median	94	83	79
	Min, Max	42.0, 160.0	40.0, 146.0	39.0, 139.0
ALT (ukat/L)	Mean (SD)	15.1 (5.32)	15.4 (9.25)	11.6 (5.06)
	Median	13	12	10.5
	Min, Max	9.0, 26.0	6.0, 32.0	6.0, 22.0
AST (ukat/L)	Mean (SD)	15.8 (6.15)	15.9 (8.23)	15.8 (5.17)
	Median	14	15	17
	Min, Max	8.0, 25.0	5.0, 30.0	9.0, 23.0
Bilirubin (umol/L)	Mean (SD)	0.4 (0.18)	0.3 (0.10)	0.3 (0.12)
	Median	0.4	0.3	0.3
	Min, Max	0.2, 0.7	0.2, 0.5	0.2, 0.6
spKt/V	Mean (SD)	NA	1.9 (0.38)	1.9 (0.36)
	Median	NA	1.9	1.8
	Min, Max	NA	1.4, 2.6	1.4, 2.4

**Table 5:** Per patient blood flow rate and dialysate flow rate in the PP population

Subject number	Blood flow rate (mL/min)	Dialysate flow rate (mL/min)
S001	450	700
S004	600	800
S008	450	600
S010	450	700
S011	400	700
S012	450	700
S013	450	700
S014	450	700
S015	450	700
S017	450	700
S020	600	800

### Characteristics and dynamics of peridialytic acetate and bicarbonate blood concentrations PP population

**Fig. 2:**
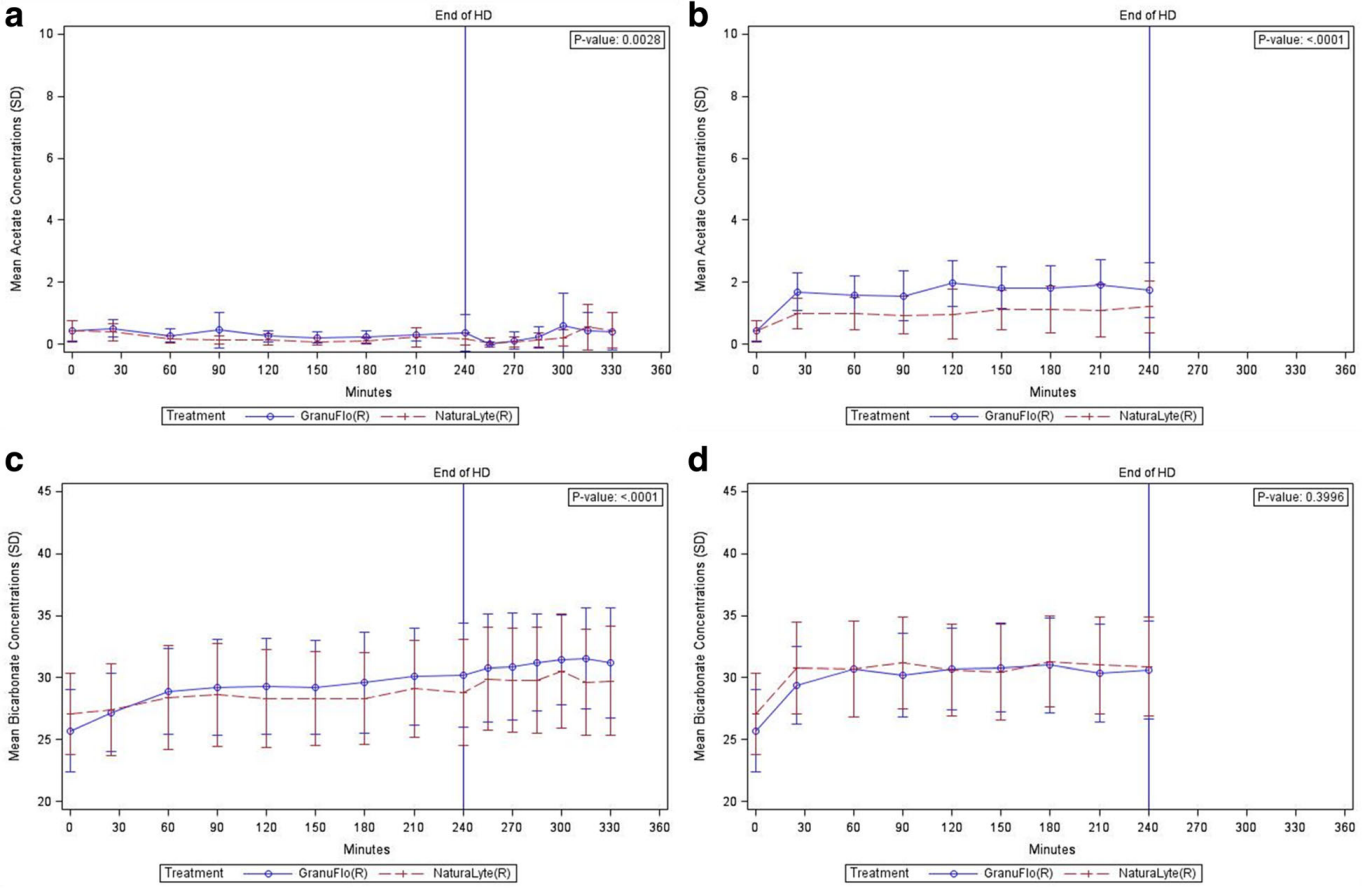
Peridialytic Blood Acetate and Bicarbonate Concentrations by Treatment Population. Mean peridialytic concentrations of acetate and bicarbonate in the arterialized and venous blood of hemodialysis patients by treatment population. **a** Acetate Concentrations (mmol/L) in Arterialized Blood vs Time by Treatment Population (±SD). **b** Acetate Concentrations (mmol/L) in Venous Blood vs Time by Treatment Population (±SD). **c** Bicarbonate Concentrations (mEq/L) in Arterialized Blood vs Time by Treatment Population (±SD). **d** Bicarbonate Concentrations (mEq/L) in Venous Blood vs Time by Treatment Population (±SD)

Linear mixed effect models with patient-specific random effects identified that longitudinal concentration trajectories of arterialized acetate during dialysis were significantly distinguishable between GranuFlo® and NaturaLyte® (*p* = 0.003, Fig. 2); however, differences at each time point were minimal and within the expected range. Standard deviations of acetate values overlap at every time point between the NaturaLyte® and GranuFlo® groups, suggesting no differences at individual time points. Longitudinal trajectories of acetate in the venous blood show that differences in acetate concentrations between acid buffer concentrates were more pronounced (*p* < 0.001, Fig. 2), as compared to arterialized blood.

Analysis of peridialytic bicarbonate concentrations demonstrated that cumulative curves for arterialized blood versus time were slightly higher with use of GranuFlo® as compared to NaturaLyte® (*p* < 0.001; Fig. 2). No difference was observed in the cumulative curves for venous blood bicarbonate concentrations versus time with use of NaturaLyte® and GranuFlo® (*p* = 0.4; Fig. 2). Arterialized blood bicarbonate concentrations increased gradually throughout dialysis in both groups. The changes were slightly larger with use of GranuFlo®: At 240 min, mean bicarbonate concentration was 30.2 (SD ± 4.16) mEq/L in GranuFlo® and 28.8 (SD ± 4.26) mEq/L in NaturaLyte® (Table 6).

**Table 6:** Peridialytic arterialized blood bicarbonate concentrations (mEq/L) summarized by time point in the PP population

Mean arterialized blood bicarbonate concentrations (±SD)		
Time point	NaturaLyte	GranuFlo
Pre-Dialysis	27.1 (3.28)	25.7 (3.33)
25 Minutes of HD	27.4 (3.69)	27.2 (3.16)
60 Minutes of HD	28.4 (4.20)	28.9 (3.48)
90 Minutes of HD	28.6 (4.14)	29.2 (3.85)
120 Minutes of HD	28.3 (3.95)	29.3 (3.89)
150 Minutes of HD	28.3 (3.80)	29.2 (3.77)
180 Minutes of HD	28.3 (3.71)	29.6 (4.09)
210 Minutes of HD	29.1 (3.90)	30.1 (3.90)
240 Minutes of HD	28.8 (4.26)	30.2 (4.16)
15 Minutes after HD	29.9 (4.18)	30.8 (4.34)
30 Minutes after HD	29.8 (4.21)	30.9 (4.28)
45 Minutes after HD	29.8 (4.26)	31.2 (3.91)
60 Minutes after HD	30.5 (4.60)	31.4 (3.64)
75 Minutes after HD	29.6 (4.27)	31.6 (4.10)
90 Minutes after HD	29.8 (4.37)	31.2 (4.44)

### Characteristics of peridialytic acetate and bicarbonate concentrations per patient

Individual peridialytic acetate and bicarbonate concentrations for each patient in the blood and dialysate are shown in Additional file 1: Figure S3 and Additional file 2: Figure S4. Arterialized blood acetate concentrations were relatively unaltered before, during and after HD with use of either acid buffer concentrate. There was a slight and stable increase of ~1 mmol/L in venous blood acetate concentrations after initiation and throughout HD. In three patients (S010, S015, S017), we did find inflow dialysate acetate concentrations were not consistent with known concentrations for the dialysate buffer types; due to this we performed an additional analysis excluding these patients, which revealed no remarkable differences in any results and trends compared to the analyses in the entire PP population (Additional files 3: Table S8 and Additional file 4: Figure S5).

Bicarbonate concentrations in arterialized blood were found to gradually increase throughout HD. Venous blood bicarbonate concentrations increased after initiation of HD and remained relatively stable throughout HD. None of the patients reached arterial bicarbonate levels greater than 40 mmol/L by the end of the hemodialysis session and the predominant driver of rise was the dialysate bicarbonate prescription, and not the acetate content in the concentrate. Further, we observed that cumulative delivery of bicarbonate via HD never exceeded the prescribed bicarbonate concentration in any patient with use of NaturaLyte® or GranuFlo®.

### Dynamics of Postdialysis bicarbonate concentrations

Investigation of the dynamics of postdialysis blood bicarbonate concentrations was performed via analyzing the change in arterialized blood bicarbonate at 240 min of HD to 90 min postdialysis, which was 0.38 mEq/L for NaturaLyte® and 0.56 mEq/L for GranuFlo® (*p* = 0.8).

### Modeling of patient bicarbonate concentrations during dialysis

Bicarbonate delivery during HD appears to be determined by the prescribed concentration. The difference in bicarbonate concentrations between dialysate inflow and the arterialized blood diminishes over time and is shown peridialytically in Additional file 2: Figure S4 as the difference between green (inflow) minus blue (arterial) lines. Notably, this difference does not become a negative value as arterialized blood bicarbonate (blue) never becomes larger than or crosses the dialysate inflow concentration. Furthermore, venous blood bicarbonate (red) is always below dialysate inflow concentration suggesting that blood returning to the patient never reaches the concentration of the bicarbonate inflow (similar to arterial acetate and inflow acetate).

Linear mixed models were constructed to model difference between bicarbonate in the dialysate inflow and arterialized blood (Table 7), and suggest the expected arterialized blood concentration can be estimated as:

**Table 7:** Estimating the Difference between Bicarbonate Dialysate Inflow and Arterial Blood Bicarbonate (mEq/L) at Any Time Point during 240 Minute Dialysis Treatment

Effect	Estimate	Standard error	*P*-Value
Intercept	17.6066	2.2332	<0.0001
Difference if GranuFlo (if NaturaLyte, zero)	−1.543	0.2609	<0.0001
Pre-dialysis Arterial Bicarbonate (mEq/L)	−0.3495	0.0782	<0.0001
Hours from the Start of Dialysis (per hour)	−0.7034	0.0893	<0.0001

Estimates are calculated using linear mixed model

$$ {\displaystyle \begin{array}{l}\left[ Dialysate Bicarbonate Inflow\right]\hbox{--} \left[ Arterialized Blood Bicarbonate\right]=\hfill \\ {} 17.61\hbox{--} 1.54\ \left[ if patient is treated with GranuFlo\circledR \left]\hbox{--} {0.35}^{\ast }\ \right[ Predialysis Arterial Bicarbonate\left]\hbox{--} {0.70}^{\ast }\ \right[ Number of Hours into the Treatment\right]+\left[ Error Term\right]\hfill \end{array}} $$

This equation can be transformed as:

$$ {\displaystyle \begin{array}{l}\left[ Arterialized Blood Bicarbonate\right]=\\ {}\left[ Dialysate Bicarbonate Inflow\right]\hbox{-} 17.61+ 1.54\ \left[ if patient is treated with GranuFlo\circledR \right]+{0.35}^{\ast }\ \left[ Predialysis Arterial Bicarbonate\right]+{0.70}^{\ast }\ \left[ Number of Hours into the Treatment\right]+\left[ Error Term\right]\end{array}} $$

For example, the modeling would suggest that the estimated difference for a patient treated with GranuFlo® who has a predialysis arterial bicarbonate concentration of 22 mEq/L (which is the reported mean of the HD population [6]) and a bicarbonate inflow of 37 mEq/L (the prescription of bicarbonate for most patients), at 4 h into the treatment would be determined by:

$$ 37\hbox{--} 17.61+ 1.54+{0.35}^{\ast }\  22+{0.70}^{\ast }\  4+\left[ Error Term\right]= 31.43+\left[ Error Term\right] $$

This means that the patient’s ending arterial bicarbonate concentration at 4 h of HD would be 31.43 mEq/L (or 5.57 mEq/L below dialysate bicarbonate inflow).

However, if we wish to estimate this difference for the same patient but treated with NaturaLyte®, we would get the following result:

$$ 37\hbox{--} 17.61+ 0+{0.35}^{\ast }\  22+{0.70}^{\ast }\  4+\left[ Error Term\right]= 29.89+\left[ Error Term\right] $$

This calculation would estimate the patient’s ending arterial bicarbonate concentration at 4 h into the treatment to be 29.89 *mEq/L (or 7.11* mEq/L below dialysate bicarbonate inflow).

The error term is the result of linear mixed effect model and the model’s aim is to find regression coefficients that minimize this error term for the population of patients.

The analysis in Table 3 shows that dialysate inflow, predialysis blood bicarbonate concentration, time on dialysis, as well as the concentrate type are associated with differing ending arterialized bicarbonate levels; patients treated with GranuFlo® have an arterialized blood bicarbonate concentration 1.54 mEq/L higher than patients treated with NaturaLyte®. Notably outputs of the models should be used within the range of values in this analysis; unusual input values may result in unlikely outputs, such as negative differences.

## Discussion

In this randomized cross-over trial examining excursions of acetate and bicarbonate concentrations, subjects tolerated the change between the two acid buffer concentrates without the occurrence of any related adverse events. We identified that a small fraction of dialysate acetate was delivered to patients during HD using NaturaLyte® or GranuFlo® acid buffer concentrates, as seen by slight increases in venous blood acetate concentrations leaving the dialyzer and returning to the patient. However, acetate that was delivered was found to be rapidly cleared from the circulation since arterialized blood acetate concentrations were relatively unchanged during and after dialysis. We believe that the small amount of acetate in the dialysate solutions used does not appear to overwhelm the liver’s capacity for metabolism. We observed bicarbonate concentrations were within and did not exceed the prescribed target range with use of both NaturaLyte® and GranuFlo® acid buffer concentrates. During dialysis there were no differences between acid buffer concentrates in the cumulative curves of venous blood bicarbonate concentrations (*p* = 0.4; Fig. 2), which indicates patients were delivered similar concentrations of bicarbonate and there were no differences in the postdialysis period, suggesting that the acid buffer concentrate type does not influence the ultimate concentration of bicarbonate. Notably, we found that there was incomplete diffusion of bicarbonate from the dialysate delivered to the patient with venous blood concentrations being considerably lower than inflow dialysate; this was also true for acetate. Overall, the concentration of acetate in NaturaLyte® and GranuFlo® acid buffer solutions did not impact the overall buffer received by patients as observed by bicarbonate levels being within the prescribed concentration with both dialysate acid buffer types, and we found that the concentrations of bicarbonate prescribed is the major determinate of buffer delivery from the dialysate. These findings show that the speculated concept of a total buffer, where the prescribed bicarbonate equals the sum of bicarbonate in the dialysate bicarbonate buffer plus the precursors in the acid buffer [20], is false. Since, all dialysate buffers use a precursor of bicarbonate for an acid to buffer the bicarbonate in the dialysate, which have the same metabolic pathway as acetate, dialysate solutions that are acetate free would be anticipated to have similar impacts on the overall bicarbonate prescription as found to have in this study, but further investigations are needed.

The normal physiological concentrations of acetate in human blood have not been well established. The reported ranges vary considerably from 0.05 mmol/L to 0.52 mmol/L in the general population [12–14], and for dialysis patients, predialysis acetate concentrations range from 0.02 mmol/L to 0.78 mmol/L [13, 15–19]. These variations are expected with the myriad of proprietary detection methods used for determining blood acetate concentrations, many of which utilized gas chromatography methods that recently have been reported to not be appropriate for quantitative measurements [20]. We used a colorimetric assay kit validated for detection of dialysate and blood acetate concentrations [9–11], and to our knowledge this is the first fully validated method in humans. However, relative normal physiological values of acetate concentration in HD patients have not been elucidated due to a lack of widespread testing. We observed predialysis acetate blood concentrations of approximately 0.5 mmol/L, which is consistent with some previous studies [16].

Currently, there are few reports that have investigated the dynamics of acetate concentrations in the blood associated with bicarbonate HD. A recent Spanish randomized clinical trial by Sánchez-Canel et al. investigated the acid-base status of 22 patients receiving hemodiafiltration (HDF) with bicarbonate dialysate containing 3 mEq/L of acetate, or acetate free biofiltration (AFB), which is a bicarbonate and acetate free dialysate technique that is combined with sodium bicarbonate infusion into the venous blood leaving the dialyzer [16]. This study found acetate concentrations rise about 0.05 mmol/L by the midpoint of HDF, are similar post-HDF, and return to baseline after HDF. As expected, acetate concentrations were unchanged during and after AFB. With both HDF and AFB the bicarbonate concentrations were not remarkably different, but repleted to a slightly greater extent with AFB. The HDF results show consistent trends with our observations for venous blood values that only a small amount of dialysate acetate is delivered to the patient and does not alter blood bicarbonate concentrations or the bicarbonate administered.

Conversely, a French study of HD patients treated with bicarbonate dialysate containing 4 mEq/L of acetate or bicarbonate dialysate buffered with hydrochloric acid reported large increases in blood acetate concentrations that contrast our findings [18]. With use of an acetate acid buffered dialysate, 78% of patients had a 10 fold increase in blood acetate concentration from predialysis to postdialysis. Surprisingly, with use of a hydrochloric acid buffered dialysate, 12% of patients had >3 fold increase in blood acetate concentration from predialysis to postdialysis.

In a historic study comparing an acetate dialysate versus an acetate acid buffered bicarbonate dialysate in the United States, it was found that blood bicarbonate concentrations decreased predialysis to postdialysis with use of acetate dialysate, while the bicarbonate concentrations rose about 6 mEq/L with use of acetate acid buffered bicarbonate dialysate [19]; this observation supports the concept that acetate delivered from the dialysate does not alter the concentration of delivered bicarbonate, which remained within the prescribed target range. The study reported that blood acetate concentrations rose by about 0.06 mmol/L by the end of HD with use of acetate acid buffered bicarbonate dialysates.

Although our results found that acetate in acid dialysate buffers do not alter the prescribed bicarbonate concentrations received from HD, it is important to denote that there is a longstanding controversy of the optimal bicarbonate prescription for physicians to utilize. The results of a DOPPS analysis suggest all-cause mortality increases by 8–9% for every 4 mmol/L increase in the prescribed concentration of dialysate bicarbonate in HD patients [21]. This risk is thought to be linked to the development of metabolic alkalosis, which is supported by findings from a recent study of Japanese patients that found that a pH >7.4 is associated with increased risks of both all-cause and cardiovascular related mortality in dialysis patients [22]. A study conducted in France on 12 patients observed that with use of a bicarbonate prescription of 38 mmol/L, there was a pH of 7.43 at the start of HD and pH of 7.51 at the end of the HD [23], which indicates there was some alkalosis associated with use of this level of bicarbonate prescription in this cohort. Despite that some reports suggest that higher bicarbonate prescriptions may lead to increased risks of negative outcomes, a recent series of literature reviews identified that after adjustment for the confounding factors of nutritional status that higher bicarbonate levels may not adversely affect patient outcomes, and that higher levels of pre-dialysis blood bicarbonate levels might indicate a need for nutritional assessments and interventions for optimizing protein intake [20, 24]. Currently, the optimal bicarbonate prescription is not known and individualized patient management is prudent at this time.

It is notable to mention that our study has several limitations that could have contributed to some of the findings. Primarily, these include no formal power calculation for determination of sample size, a small patient number, and short study duration. A formal sample size calculation was not performed due to there not being any been previous studies that have been performed with a validated acetate assay to provide the necessary reference value and variations for acetate blood levels to base an estimation upon. Despite a small patient number, the crossover design and high number of collection time points (84 data points per patient per treatment and 1680 data points to calculate the main study endpoints) made the statistics in this study strong and the data had low variability. Overall, this allowed for sound conclusions to be drawn based on both single and multiple time point comparisons. Although the duration of the trial was only 2 weeks, this intensive interrogation of the metabolic dynamics of levels of short lived analytes (acetate and bicarbonate) should not require a long term study.

Another limitation of this study is that it was only single blinded, and not double blinded. However, with the laboratories and patients being blinded from dialysate assignment in this investigation that assessed changes in laboratory values, the parties that we thought could have potentially influenced the results were blinded. Also, we did not study acetate-free dialysate types and the metabolic differences remain uncharacterized.

An additional limitation of the study was that the investigation was performed on a patient population that is not consistent with the average HD population in the United States. The patients in this study were majorly of African American race (81.8%) and were on average about 4 years younger and had a BMI that was 4 kg/m^2^ higher than the general dialysis population in the United States [25, 26]. Nevertheless, the patient population resembles the dialysis patient population in the geographical area (USRDS geographical network 8) that the study was conducted. Dialysis patients in this area typically are 60.6% of African American race and have a mean age of 55.1 year old [25]. Further, a lower percent of patients in this study were diabetic (40%) compared to overall rate of 63% in the population of patients at Fresenius Medical Care North America. Although there are some differences in the study population versus the general dialysis population, the overall intent was not to match the general population for generalizability, but to have internal validity in determining the changes in peridialytic acetate and bicarbonate levels in humans using two acid dialysate types containing different acetate concentrations to buffer the bicarbonate dialysate during treatment.

An additional limitation is that we did not capture information or adjust for the patients’ lean body mass, and since animal studies have indicated that acetate may be metabolized in the muscle [27]. We did measure BMI, however, it was decided to not adjust our analysis since this body composition measure is considered to be inaccurate in patients with ESRD that are known to have volume overload and muscle wasting affecting the metric [28]. With time changes in fat and/or muscle mass may alter acetate metabolism, however, with a cross over study design, every patient was her/his own control, so the lean body mass of the patient was expected to remain relatively constant for the 2 week duration of the trial. There is a need for further long-term trials that investigate acetate metabolism adjusting for body composition using methods bioelectrical impedance vector analysis to quantify lean muscle mass.

Furthermore, we’d like to note that this study was conducted using traditional diffusion based hemodialysis treatments using high-flux dialyzers. As such, we cannot generalize these findings to convective hemodiafiltration therapy. Several studies suggest that acetate-free dialysis may be preferred in hemodiafiltration treatments [29–31], and in vitro studies in endothelial cell cultures suggest that acetate-free bicarbonate solutions may be more favorable with respect to the cell-to-cell influences on acid-base physiology in one tissue type [32, 33].

Despite the limitations, this study does have the strengths of being a randomized trial of stable HD patients that did not have any common clinical abnormalities that could alter acetate and/or bicarbonate metabolism and dietary intake was controlled proximal to study treatments. Additionally, this study provides a comprehensive profile of the dynamics of peridialytic concentrations of acetate and bicarbonate in both the arterialized and venous blood and dialysate of HD patients, which has not been investigated previously, and current reports do not define if arterialized or venous blood was used for analyses. Additional studies to further establish physiologic dynamics and determine the normal values of acetate are needed, and after this, the characterization of the transmembrane kinetics of acetate across the dialyzer membrane using mass transfer calculations will be of importance.

## Conclusions

In conclusion, the results of this clinical trial indicate that acetate concentrations in NaturaLyte® and GranuFlo® acid buffer concentrates do not cause excursions in blood bicarbonate concentrations that were above the prescribed level in patients during HD or in the postdialysis period. These findings suggest that acetate delivered to the patient from the dialysate is rapidly metabolized by the body, but has a minor impact on blood bicarbonate levels during or after HD. There is incomplete diffusion of both bicarbonate and acetate delivered from the dialysate to the blood. The concept of a “total buffer”, which suggests acetate is directly metabolized in a 1:1 ratio into additional bicarbonate yielding significantly higher blood bicarbonate concentrations, did not appear to exist. As compared to the NaturaLyte® acid buffer, use of GranuFlo® is associated with a slightly faster reduction in the arterialized blood to inflow dialysate concentration gradient of bicarbonate during the course of a HD treatment.

## Additional files

Additional file 1: Figure S3. Patient Peridialytic Acetate Concentrations in the Blood and Dialysate by Acid Concentrate Type. Peridialytic acetate concentrations for each patient in the blood and dialysate by acid concentrate type. 3.1: Individual Acetate Concentrations vs Time NaturaLyte® Population. 3.2: Individual Acetate Concentrations vs Time GranuFlo Population. (DOCX 763 kb)

Additional file 2: Figure S4. Patient Peridialytic Bicarbonate Concentrations in the Blood and Dialysate by Acid Concentrate Type. Peridialytic bicarbonate concentrations for each patient in the blood and dialysate by acid concentrate type. 4.1: Individual Bicarbonate Concentrations vs Time NaturaLyte Population. 4.2: Individual Bicarbonate Concentrations vs Time GranuFlo Population. (DOCX 675 kb)

Additional file 3: Table S8. Estimating the Difference between Bicarbonate Dialysate Inflow and Arterial Blood Bicarbonate (mEq/L) at Any Time Point during 240 Minute Dialysis Treatment - Excluding Subjects S010, S015, S017. (DOCX 12 kb)

Additional file 4: Figure S5. Mean peridialytic concentrations of acetate and bicarbonate in the arterialized and venous blood of hemodialysis patients by treatment population excluding subjects S010, S015, S017. 5.1: Acetate Concentrations (mmol/L) in Arterialized Blood vs Time by Treatment Population (±SD). 5.2: Acetate Concentrations (mmol/L) in Venous Blood vs Time by Treatment Population (±SD). 5.3: Bicarbonate Concentrations (mEq/L) in Arterialized Blood vs Time by Treatment Population (±SD). 5.4: Bicarbonate Concentrations (mEq/L) in Venous Blood vs Time by Treatment Population (±SD). (DOCX 448 kb)

## Abbreviations

AFB: Acetate free biofiltration
AVF: Arteriovenous fistula
AVG: Arteriovenous graft
ESRD: End stage renal disease
FMC: Fresenius Medical Care
GLP: Good Laboratory Practice
HD: Hemodialysis
HDF: Hemodiafiltration
IRB: Institutional Review Board
KDOQI: Kidney Disease Outcomes Quality Initiative
