# Supplementary figures and images for: The dynamics of the metabolism of acetate and bicarbonate associated with use of hemodialysates in the ABChD trial: a phase IV, prospective, single center, single blind, randomized, cross-over, two week investigation
Source: BMC Nephrol. 2017 Aug 29;18:273. doi: 10.1186/s12882-017-0683-6 (PMC5576126; doi:10.1186/s12882-017-0683-6)

| **Figure 3.1:** | **Figure 3.2:** |
| --- | --- |
| 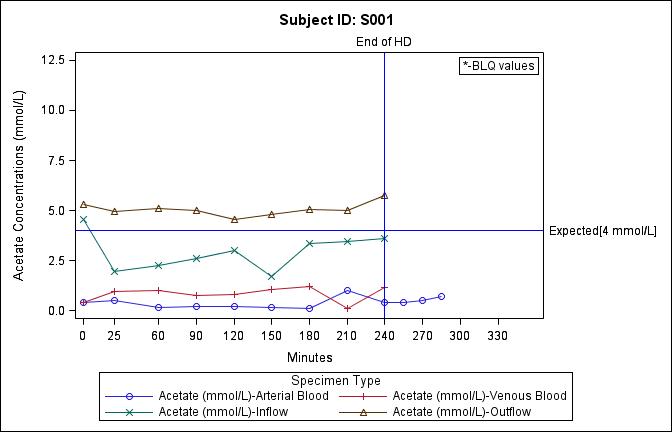 |  |
| 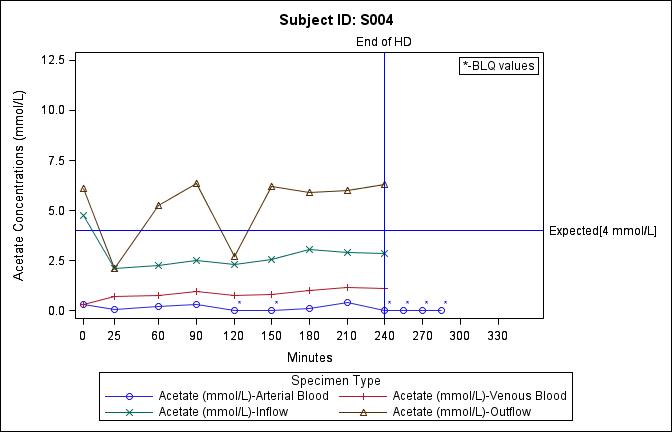 | 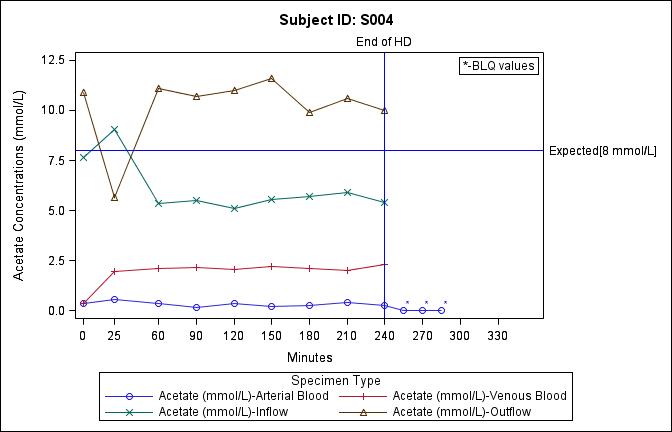 |
| 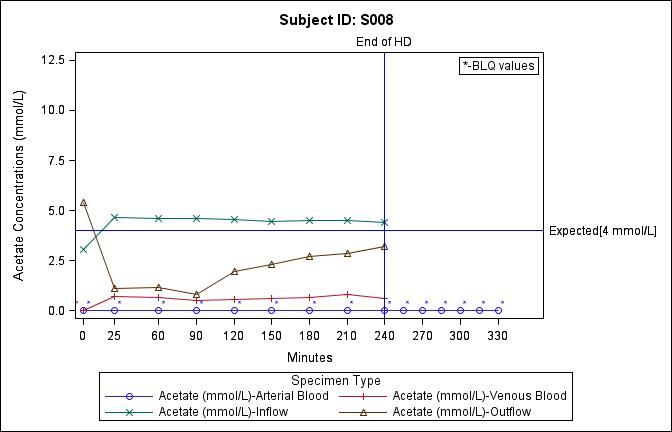 | 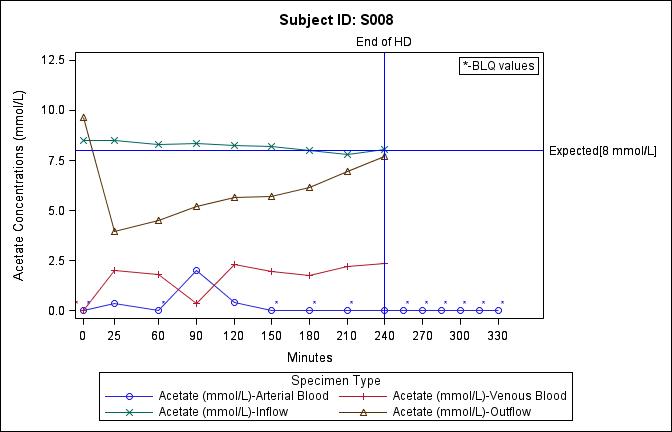 |
| 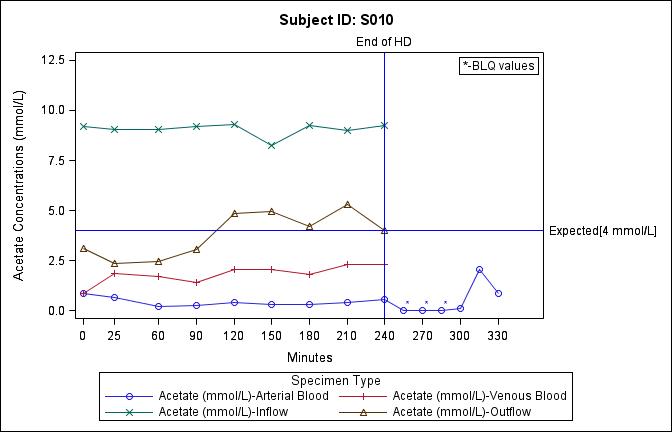 | 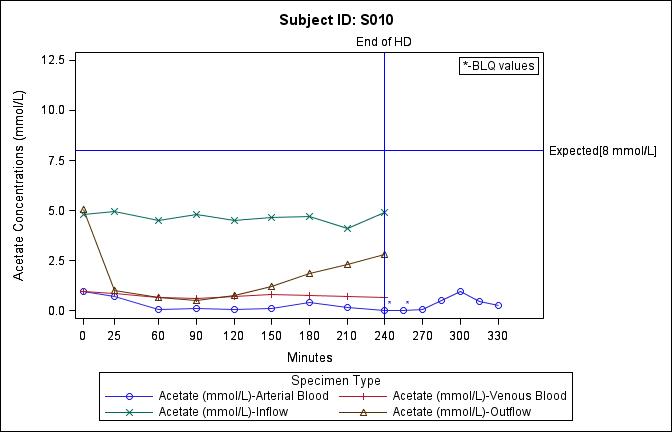 |
| 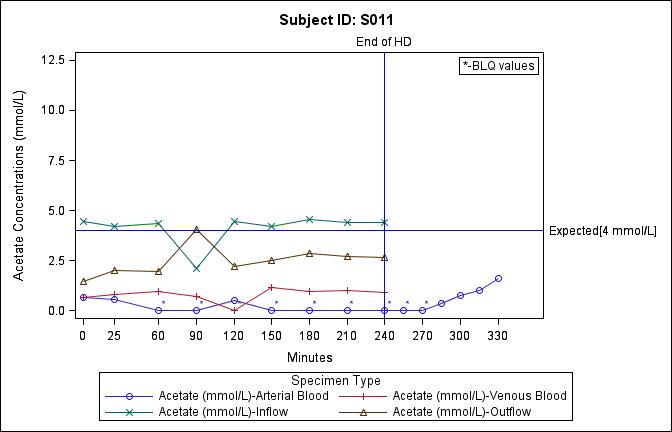 | 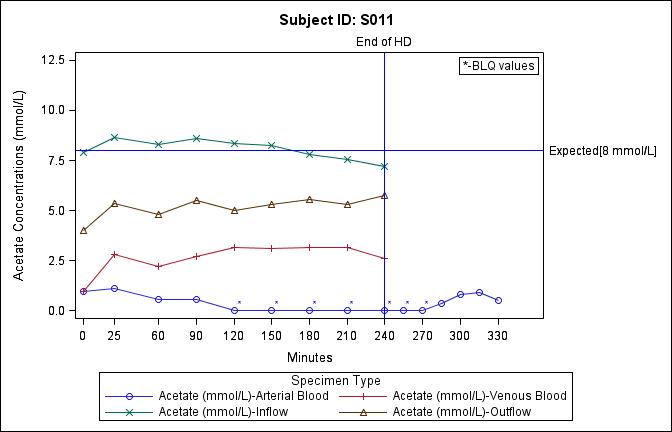 |
| 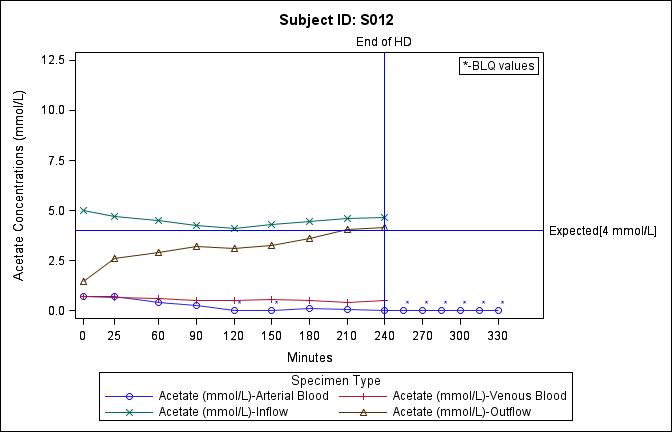 | 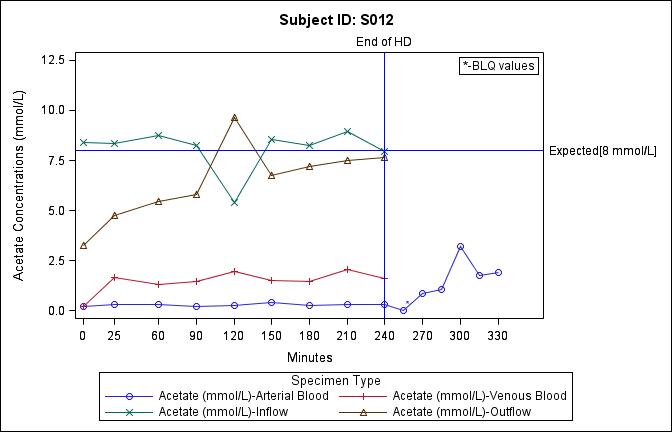 |
| 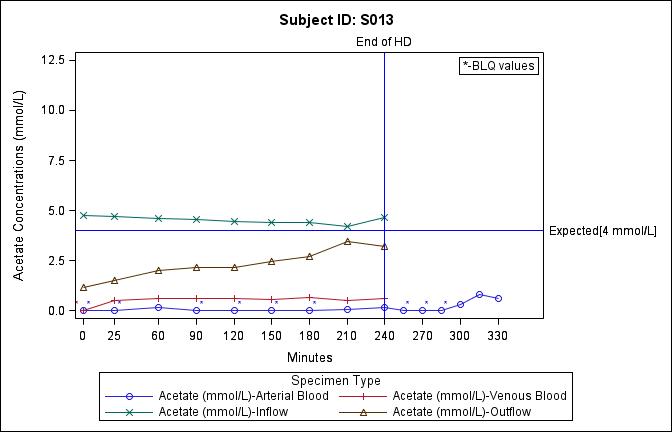 | 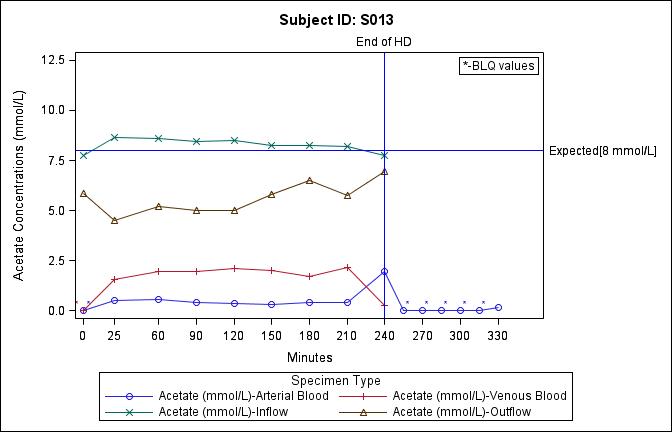 |
| 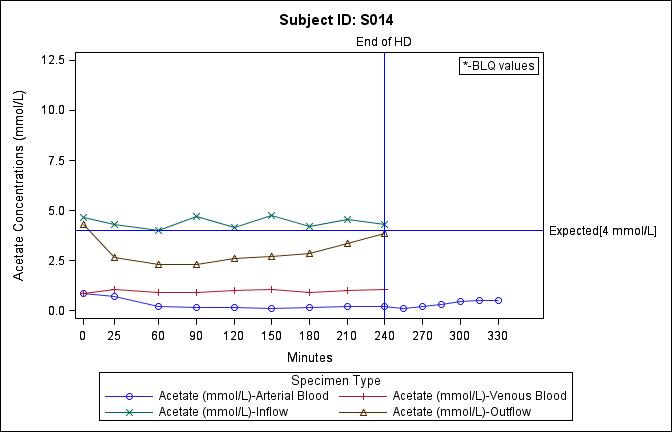 | 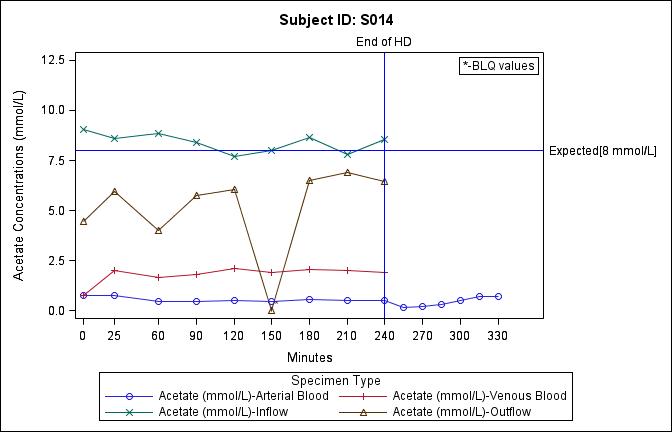 |
| 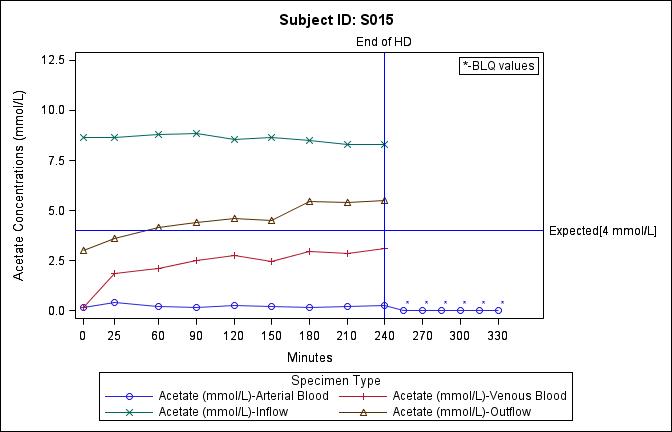 | 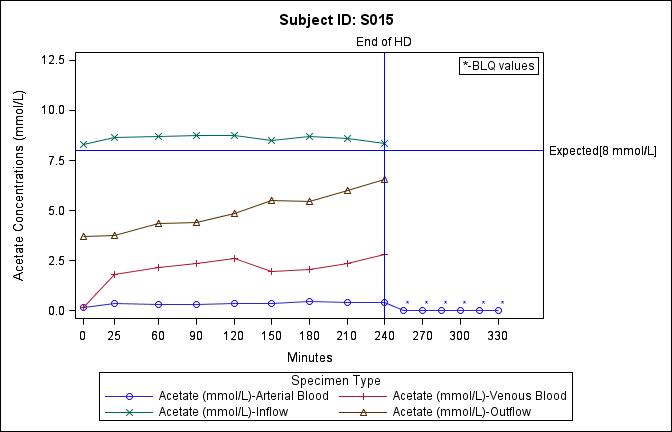 |
| 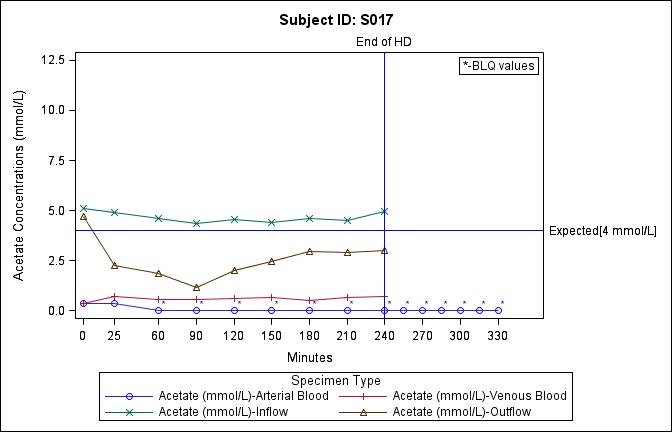 | 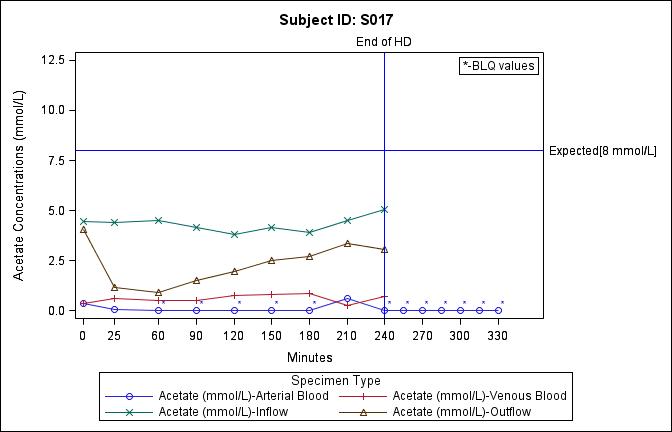 |
|  | 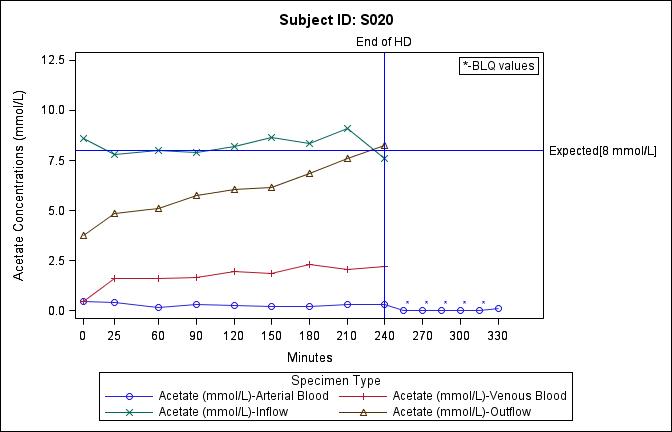 |

Supplement: Supplementary file 1 — Patient Peridialytic Acetate Concentrations in the Blood and Dialysate by Acid Concentrate Type. Peridialytic acetate concentrations for each patient in the blood and dialysate by acid concentrate type. 3.1: Individual Acetate Concentrations vs Time NaturaLyte® Population. 3.2: Individual Acetate Concentrations vs Time GranuFlo Population. (DOCX 763 kb) [file 12882_2017_683_MOESM1_ESM.docx]

| **Figure 4.1:** | **Figure 4.2:** |
| --- | --- |
| 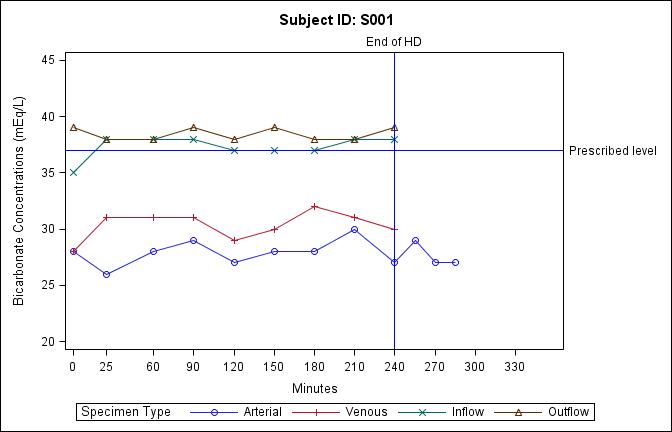 |  |
| 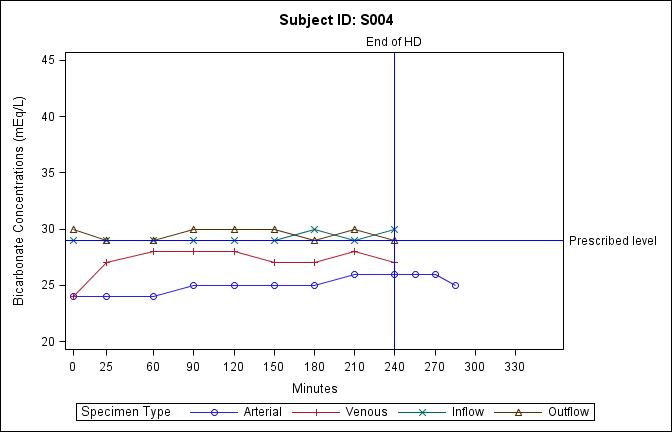 | 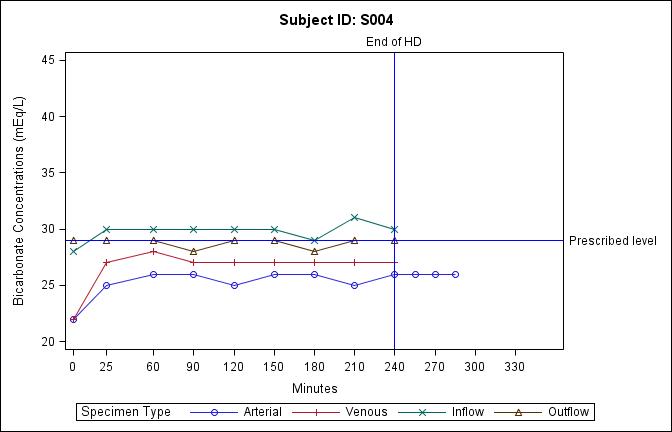 |
| 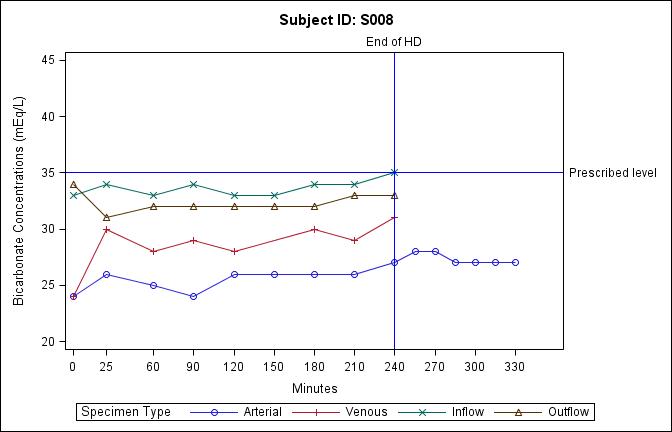 | 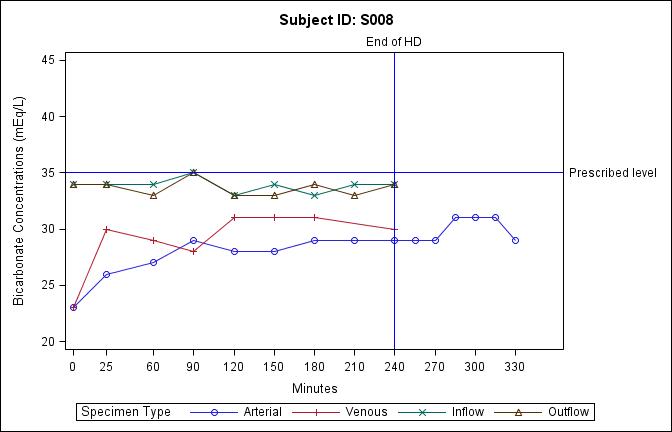 |
| 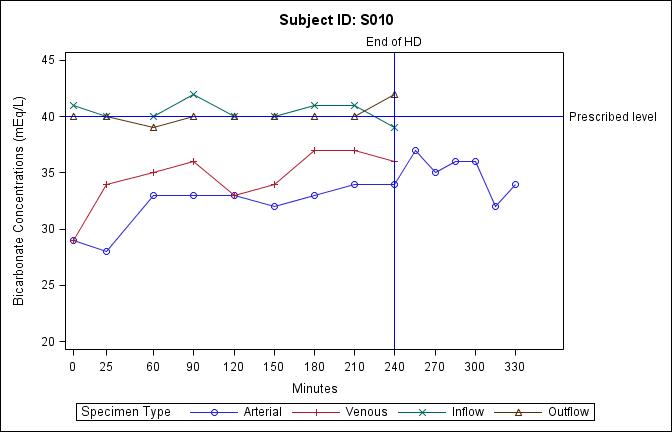 | 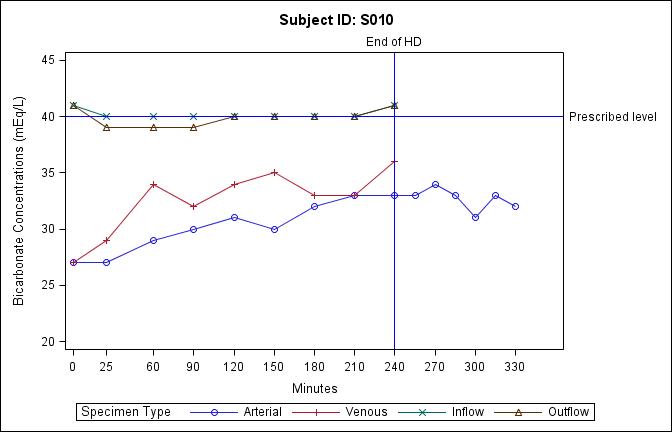 |
| 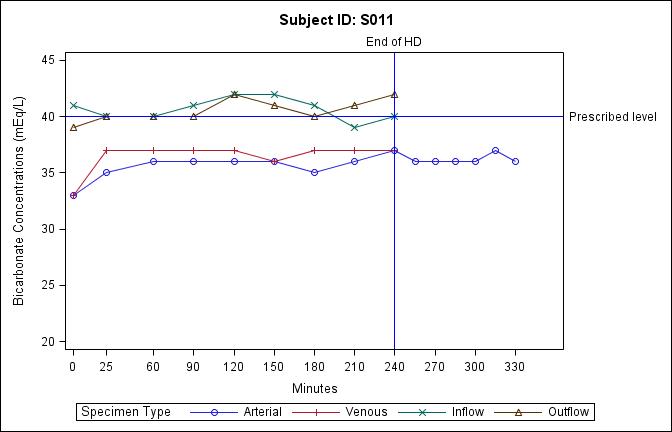 | 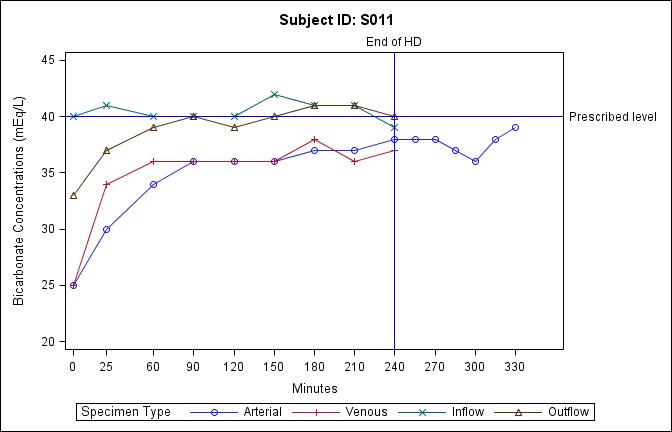 |
| 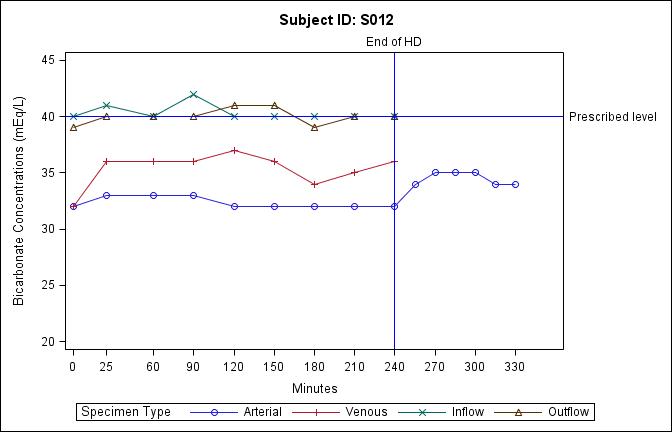 | 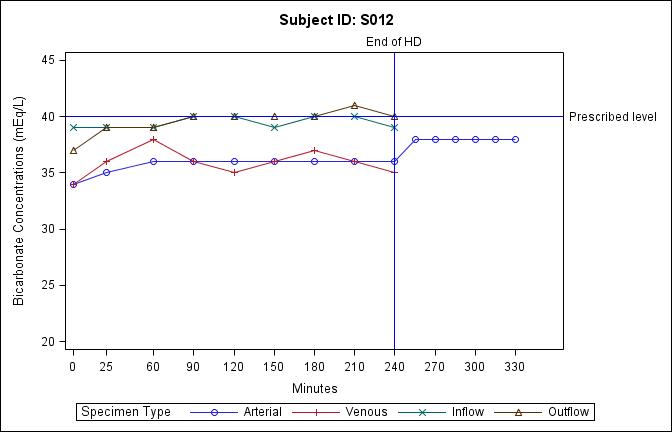 |
| 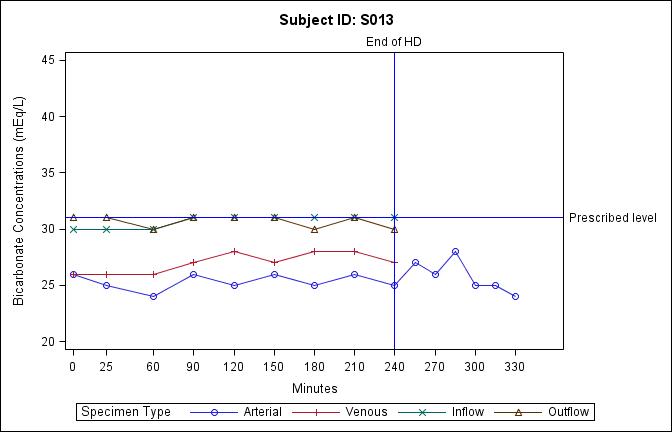 | 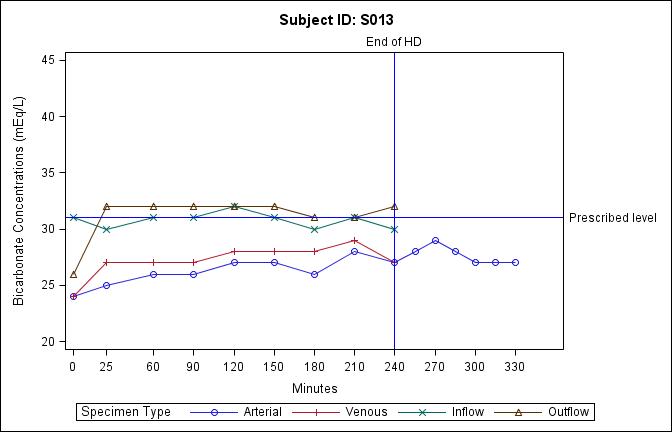 |
| 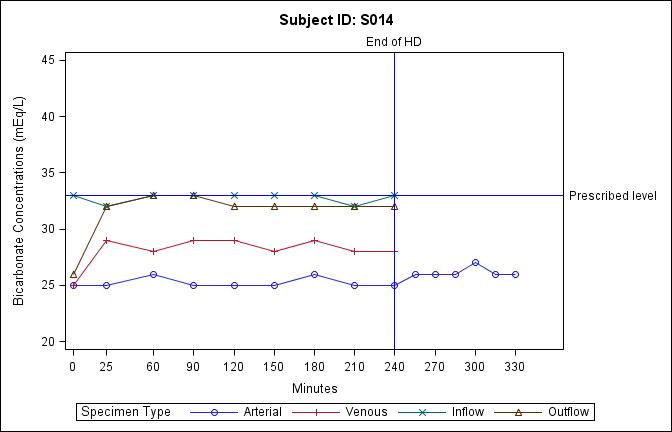 | 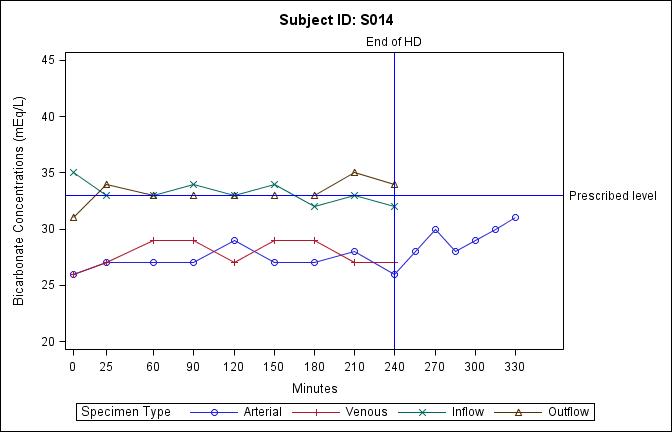 |
| 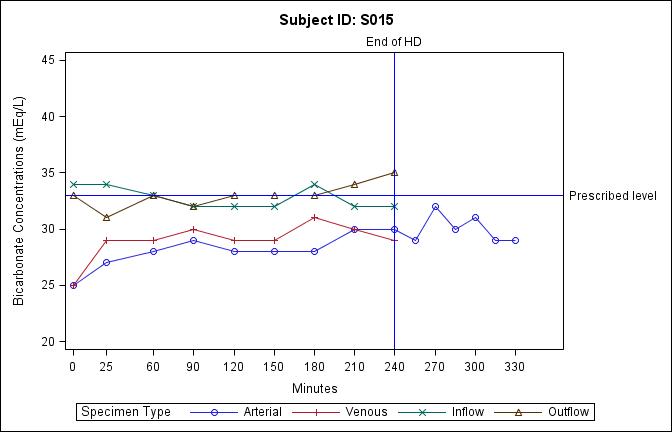 | 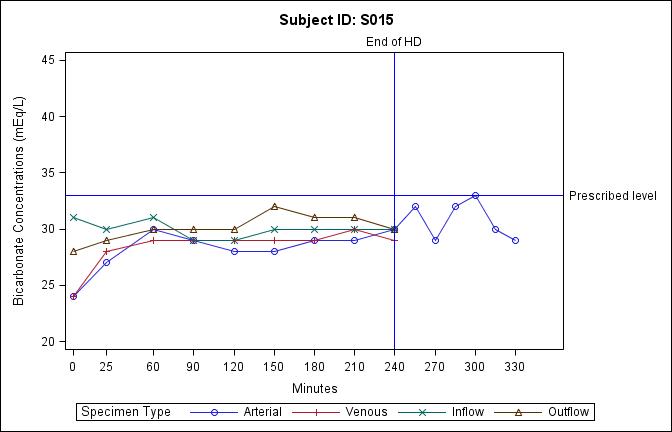 |
| 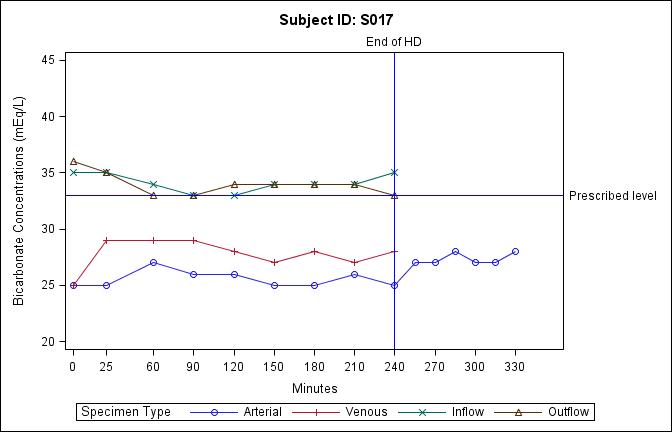 | 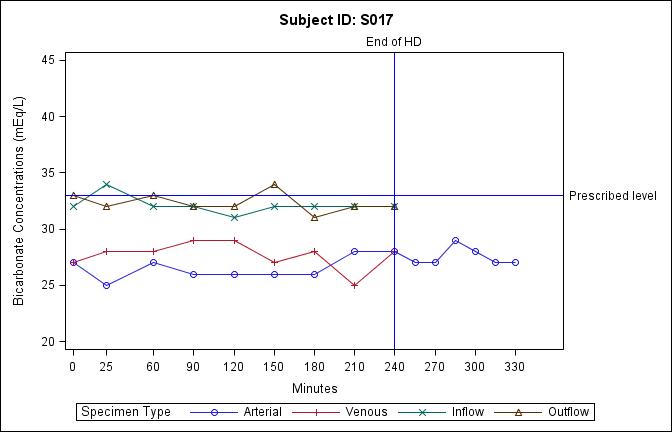 |
|  | 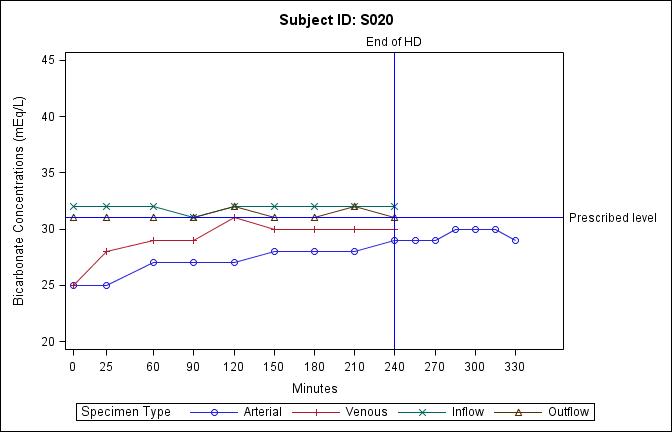 |

Supplement: Supplementary file 2 — Patient Peridialytic Bicarbonate Concentrations in the Blood and Dialysate by Acid Concentrate Type. Peridialytic bicarbonate concentrations for each patient in the blood and dialysate by acid concentrate type. 4.1: Individual Bicarbonate Concentrations vs Time NaturaLyte Population. 4.2: Individual Bicarbonate Concentrations vs Time GranuFlo Population. (DOCX 675 kb) [file 12882_2017_683_MOESM2_ESM.docx]
