# Supplementary material for: The dynamics of the metabolism of acetate and bicarbonate associated with use of hemodialysates in the ABChD trial: a phase IV, prospective, single center, single blind, randomized, cross-over, two week investigation
Source: BMC Nephrol. 2017 Aug 29;18:273. doi: 10.1186/s12882-017-0683-6 (PMC5576126; doi:10.1186/s12882-017-0683-6)
Supplement: Supplementary file 3 — Estimating the Difference between Bicarbonate Dialysate Inflow and Arterial Blood Bicarbonate (mEq/L) at Any Time Point during 240 Minute Dialysis Treatment - Excluding Subjects S010, S015, S017. (DOCX 12 kb) [file 12882_2017_683_MOESM3_ESM.docx]

**Table 8: Estimating the Difference between Bicarbonate Dialysate Inflow and Arterial Blood Bicarbonate (mEq/L) at Any Time Point during 240 Minute Dialysis Treatment - Excluding Subjects S010, S015, S017**

| **Effect** | **Estimate** | **Standard Error** | **P-Value** |
| --- | --- | --- | --- |
| Intercept | 16.7214 | 2.0827 | 0.0001 |
| Difference if GranuFlo (if NaturaLyte, zero) | -1.6437 | 0.2703 | <.0001 |
| Pre-dialysis Arterial Bicarbonate (mEq/L) | -0.3274 | 0.0727 | <.0001 |
| Hours from the Start of Dialysis (per hour) | -0.5427 | 0.0962 | <.0001 |
| Estimates are calculated using linear mixed model | | | |
