# Supplementary material for: The dynamics of the metabolism of acetate and bicarbonate associated with use of hemodialysates in the ABChD trial: a phase IV, prospective, single center, single blind, randomized, cross-over, two week investigation
Source: BMC Nephrol. 2017 Aug 29;18:273. doi: 10.1186/s12882-017-0683-6 (PMC5576126; doi:10.1186/s12882-017-0683-6)

Figure 5.1:

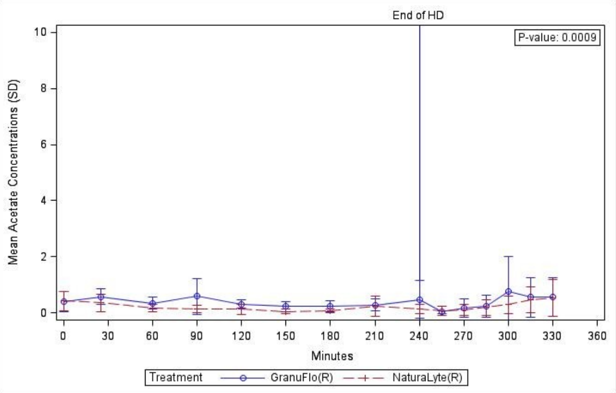

Figure 5.2:

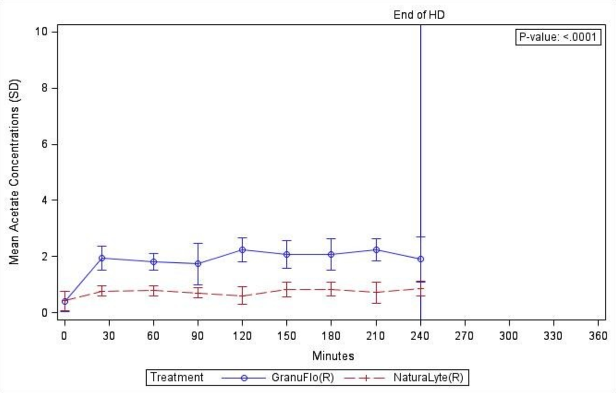

Figure 5.3:

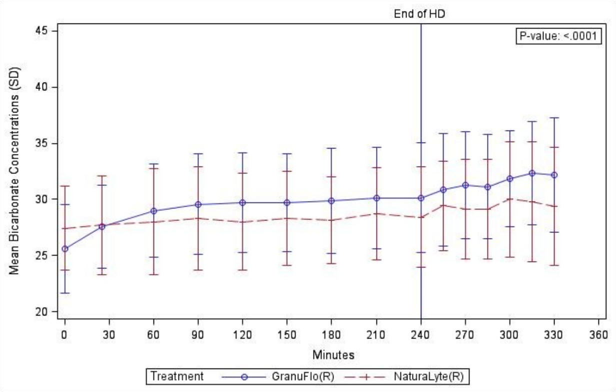

Figure 5.4:

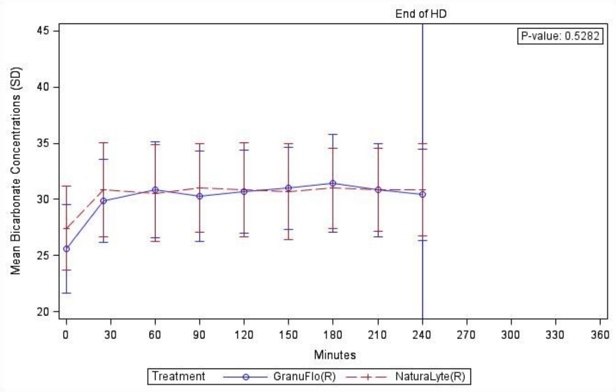

Supplement: Supplementary file 3 [file 12882_2017_683_MOESM4_ESM.docx]
